# Shear Connections Between LVL Slabs and GLT Beams: Experimental and Numerical Study

**DOI:** 10.3390/ma19214760

**Published:** 2026-11-09

**Authors:** Marcin Chybiński, Łukasz Polus, Robert Studziński

**Affiliations:** Institute of Building Engineering, Faculty of Civil and Transport Engineering, Poznan University of Technology, Piotrowo 5 Street, 61-138 Poznan, Poland; lukasz.polus@put.poznan.pl (Ł.P.); robert.studzinski@put.poznan.pl (R.S.)

**Keywords:** timber–timber composite structures, laminated veneer lumber (LVL), glued-laminated timber (GLT), shear connection, push-out test, discrete connections, numerical models

## Abstract

**Highlights:**

- Tested hexagon-head screw configurations exhibited higher stiffness and load-carrying capacity.
- Tested carpenter screw configurations showed larger ultimate slip and greater ductility.
- Load–slip curves provide input data for FEM simulations of timber–timber composite beams.

**Abstract:**

Sustainable timber–timber composite floors can facilitate CO_2_ reduction. The structural behaviour of composite floors strongly depends on the load-carrying capacity and stiffness of shear connections. For this reason, this investigation characterises the behaviour of timber–timber composite screwed connections. Push-out tests were conducted on twelve samples, each consisting of a glued-laminated timber beam, laminated veneer lumber panels, and screws. The effects of screw type and penetration depth on connection load–slip behaviour were analysed in terms of load-carrying capacity, slip modulus, and failure mode. The laminated veneer lumber panels in the numerical models were connected with the glued-laminated timber beams using discrete connections, the behaviour of which was described using the three-linear load–slip models. The push-out FE simulations were used to verify and calibrate the experimentally derived tri-linear spring characteristics intended for subsequent numerical analyses of timber–timber composite structural members. The numerical model was therefore focused on reproducing the global connection response rather than the detailed local mechanics of individual fasteners.

## 1. Introduction

In recent years, timber and timber-based composite structures have gained increasing importance, driven both by the growing emphasis on sustainable development and the continuous advancements in material performance and structural design. The connections between timber elements, as well as those between timber and concrete or steel elements, play a crucial role in these systems. Their mechanical properties—such as stiffness, capacity, and deformability—have a significant impact on the overall structural behaviour of the composite elements under service and on the verification of their ultimate limit states.

The paper [1] synthesises the findings from recent experimental investigations and analytical models, focusing on the effects of the screw type and inclination angle on the structural performance of timber–concrete composite (TCC) systems, including both connection and beam specimens, under ultimate and serviceability limit states. The authors of the cited study placed particular emphasis on the shear strength, stiffness, and long-term behaviour of “cross-pair” connections—i.e., connectors inclined with respect to the shear plane, where both compression and tension develop—as well as of connections oriented perpendicularly to the shear plane (subjected primarily to shear). The article therefore provides a comprehensive summary of the current state of knowledge regarding connections in TCC systems, which can serve as a valuable reference for practical design applications. In the study [2], the application of self-tapping screws (STS) in timber–timber composite (TTC) systems was investigated. The problem was addressed using advanced 3D brick finite element models. Numerical simulations were carried out, employing cohesive zone modelling and damage constitutive material models. The numerical analyses referred to both conventional push-out test setups (also describe in [3]) and full-scale bending configurations. In the relevant literature, push-out tests are also referred to as double lap shear tests, and have been used to investigate the behaviour of joints subjected to shearing [4,5]. Importantly, the authors demonstrated that simplified methods only approximately capture the real behaviour of full-scale assemblies and tend to significantly underestimate it. They also found that in reality the local stiffness of individual STS can be up to twice as high, highlighting the need for more accurate calculations. In the study [6], the authors addressed the issue of connections in TTC elements joined using screws or 12 mm steel dowels. The numerical analyses were supported by experimental investigations. The paper presents the results from both small-scale push-out tests and large-scale four-point bending tests of 6 m span elements. In the push-out experiments, the composite elements consisted of cross-laminated timber (CLT) and laminated veneer lumber (LVL) panels. The authors demonstrated that the constitutive material models adopted in the numerical analyses accurately reproduced the experimental results. In the study [7], the issue of connections in TTC structures was further discussed to include aspects related to sustainable building. The authors in [7] emphasised the necessity of diversifying the sources of timber supply and highlighted the high potential of beech as a raw material for the production of glulam beams. The research involved 20 double lap shear tests. The authors proposed a low-engineered floor configuration consisting of beech planks connected to a beech glulam beam using inclined screws. This solution was deemed suitable for low- to medium-span residential composite floors, particularly when seeking to establish a short supply chain of locally grown hardwood. Disassembly and reuse in timber structures are important trends [8,9]. In the study [10], the authors presented the results of experimental push-out tests that accounted for the duration of loading—an important parameter for timber structures, which are inherently susceptible to creeping [11,12,13]. The research programme focused on determining the shear capacity of the connectors joining glued-laminated timber (GLT) to cross-laminated timber (CLT), considering both adhesive and non-adhesive joints, as well as the orientation of connectors with respect to the direction of long-term loading (420 days), i.e., lengthwise or crosswise. It was found that the creep coefficient ranged from 1.05 to 3.90 and that the orientation of the CLT slab had only a minor impact on the creep behaviour, as opposed to the slab thickness, which had a major impact. An interesting observation made by the authors was that approximately 50% of the total creep deformation of TTC joints occurred within the first year of sustained loading. The continuation of research on rheological effects can be found in the study [14], which investigated the long-term behaviour of steel–timber composite (STC) connections using both experimental and numerical approaches. Over a 16-month testing time, twelve specimens with different shear connector types and CLT configurations were examined under sustained loading, while ambient humidity and temperature were continuously monitored. A rheological model accounting for moisture variation, creep, mechanosorption, and inelastic shrinkage was developed and validated against experimental data. The results showed that bolt-in-grout-pocket connectors exhibited the lowest creep coefficients and the highest long-term stiffness, whereas screw and post-tensioned connectors experienced significant stiffness reductions with increasing load levels. The authors emphasised the need for extended experimental studies to establish more comprehensive design provisions for predicting the long-term deformation and creep behaviour of STC connections under variable environmental conditions. On the contrary to long-term loading case, the study [15] investigated the early-age behaviour of screw shear connections in TCC structures through 18 push-out tests using lightweight concrete of various curing ages (12 h to 28 days). The results showed that lightweight concrete with expanded shale develops strength rapidly within the first week, in agreement with Eurocode 2 [16] predictions. Similarly, the stiffness and shear strength of screw connectors increased significantly during early curing, reaching nearly full performance after 7 days. It was found that once the concrete gains sufficient strength, the properties of the glulam and screw become the main factors governing connection behaviour. Eurocode 5 [17] was shown to underestimate the ultimate shear capacity due to its conservative treatment of the rope effect, although its prediction of slip modulus remained accurate. Finally, the proposed analytical models effectively captured the early-age mechanical response of screw connections, providing valuable insight for assessing TCC systems during the construction phase. Connectors in composite elements are evaluated not only through push-out tests but also by testing composite beam elements under bending. In the study [18], such investigations were conducted, including both laboratory experiments and numerical simulations. Four-point bending tests were performed on simply supported laminated timber beams with different connection types: rigid adhesive bonding, elastomeric adhesive bonding, vertical and inclined screw-laminated joints, and beams without bonding. The outcomes from experimental testing, analytical calculations, and numerical simulations were analysed and compared. The findings revealed that the flexural stiffness of beams with inclined screw-laminated connections and those bonded with elastomeric adhesive were comparable, both showing up to twice the stiffness of beams with vertical screw-laminated joints. Meanwhile, beams bonded with rigid adhesive demonstrated approximately 50% greater stiffness than the two previously mentioned configurations.

The reviewed above studies demonstrate significant advancements in understanding the mechanical and long-term behaviour of connections in composite timber systems. The research covers a broad spectrum—from short-term push-out and bending tests to long-term creep analyses—encompassing various connection types (screws, dowels, adhesives) and composite elements (timber–timber, timber–concrete, and steel–timber composite elements). The key findings highlight the strong influence of connector inclination, adhesive rigidity, and environmental conditions on stiffness, shear capacity, and durability. Furthermore, both experimental and numerical approaches have been shown to effectively capture the complex rheological and mechanical behaviour of such systems.

In response to the current state of knowledge, this study presents an experimental investigation based on push-out tests. Two LVL slab configurations—LVL X and LVL R—were examined, in combination with two types of screws: carpenter screws and hexagon-head wood screws. The experimental programme aimed to analyse the failure mechanisms and to draw conclusions concerning the mechanical behaviour of the tested connections. Based on the laboratory tests, tri-linear load–slip models were developed for the analysed connections and implemented as discrete spring connectors in numerical models of the push-out tests. The numerical analyses were used primarily to verify and calibrate these experimentally derived connector characteristics for their subsequent application in more complex TTC models, such as full-scale composite beams. Accordingly, the numerical study was not intended to reproduce the detailed local mechanics of individual screw–timber interactions.

## 2. Materials and Methods

### 2.1. Laminated Veneer Lumber

Two types of LVL slabs were used—LVL X and LVL R. In LVL X slabs, 20% of veneers were crossbanded, whereas in LVL R slabs, the veneers were glued together longitudinally. The thickness of the LVL X and LVL R slabs was 45 mm and 75 mm, respectively. The 45 mm and 75 mm slabs were made from 15 and 25 veneer layers of Scots pine (*Pinus sylvestris* L.) and Norway spruce (*Picea abies* L. H. Karst), respectively. The LVL panels were 500 mm long and 370 mm wide, and they were loaded in the direction parallel to the grain of each veneer in LVL R, and of most veneers in LVL X. The mechanical properties (nominal values) of the LVL panels are presented in Table 1. All elements of each timber product used in the experimental programme originated from the same respective material batch and were stored prior to testing under controlled indoor conditions corresponding to Service Class 1 conditions. Moisture content and actual density were not measured individually for the tested specimens.

### 2.2. Glued-Laminated Timber

The timber beams of 200 mm × 300 mm × 650 mm dimensions were made of GL 28. The nominal values of the mechanical properties of GL 28 are presented in Table 2.

### 2.3. Screws

**Figure 1:**
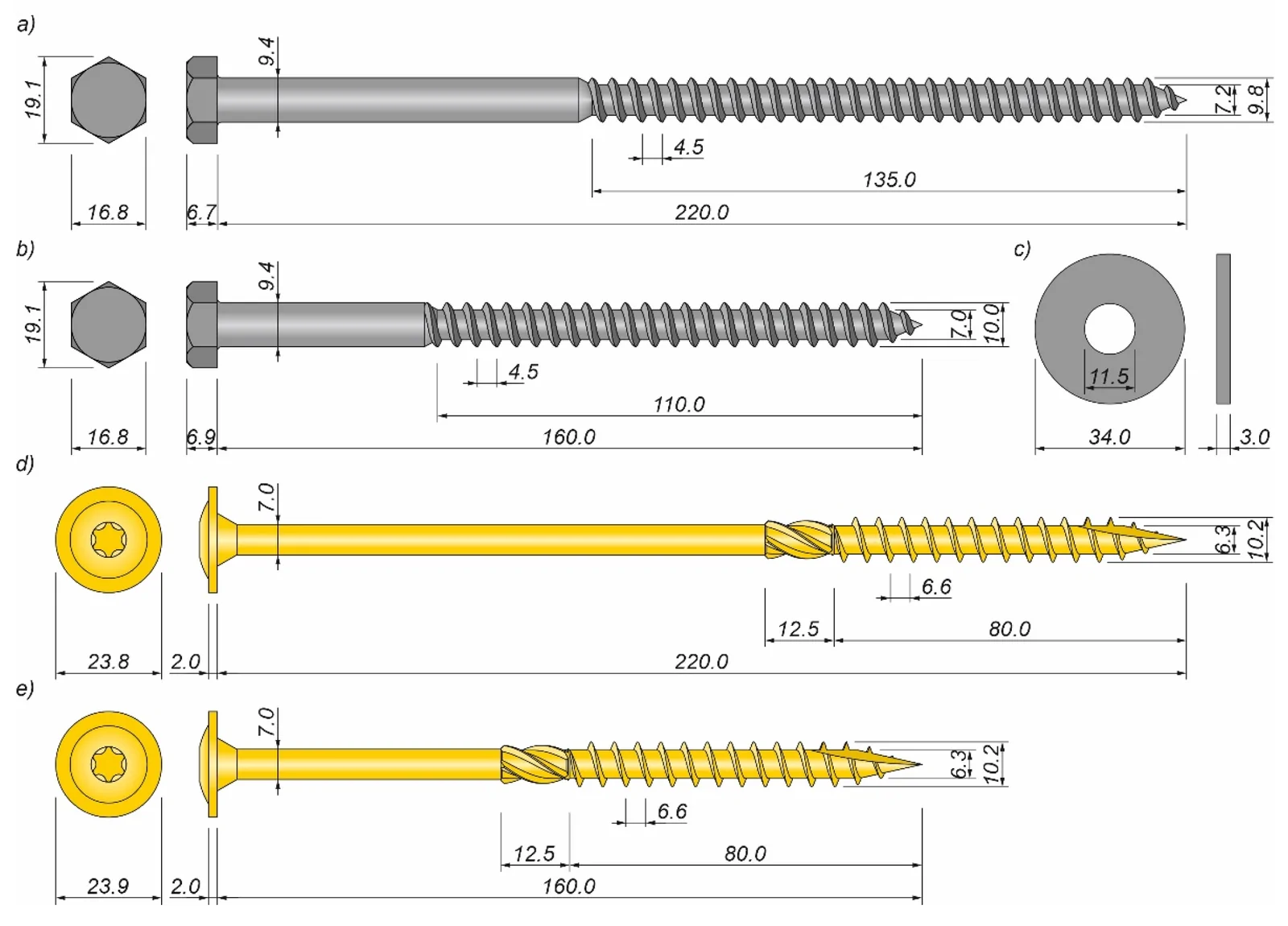
The types of screws: (**a**) hexagon-head wood screws 10 × 220; (**b**) 10 × 160; (**c**) screw washer; (**d**) carpenter screws 10 × 220; (**e**) 10 × 160 (average values).

Two types of screws were used in the fabrication of the push-out specimens, i.e., DMX carpenter and hexagon-head wood screws (Figure 1). The DMX carpenter screws (DOMAX CT) had a diameter of 10 mm and a length of 160 or 220 mm. The carpenter screws had a yield strength of 923 MPa and an ultimate tensile load of 32.0 kN [22]. The hexagon-head wood screws were made of Grade 5.8 steel with an ultimate tensile strength of 500 MPa, and a yield strength of 400 MPa. They had a diameter of 10 mm and a length of 160 mm or 220 mm (length without the screw head).

### 2.4. Push-Out Tests

**Figure 2:**
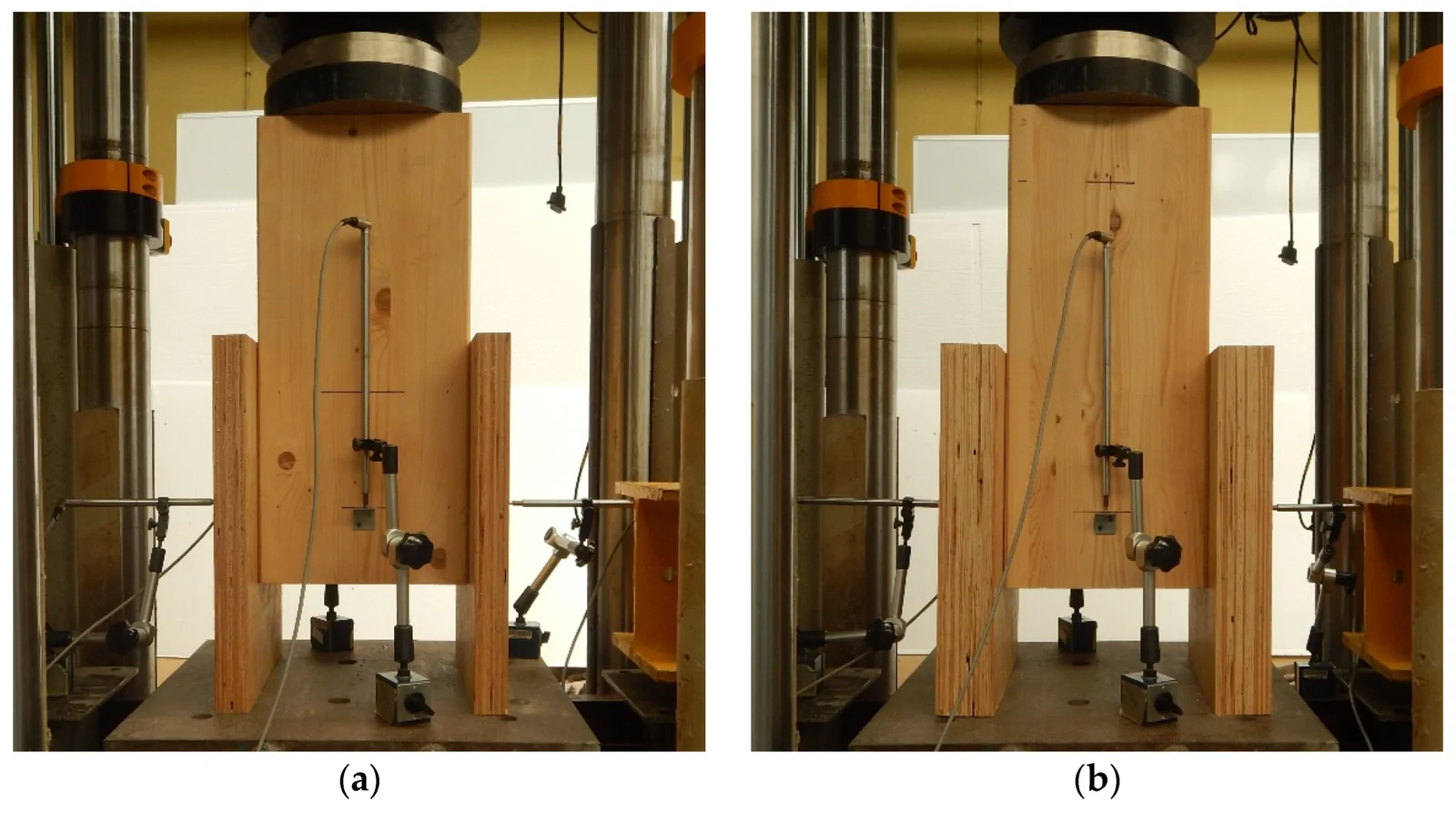
Tested specimens with: (**a**) 45 mm LVL X slabs; (**b**) 75 mm LVL R slabs.

**Figure 3:**
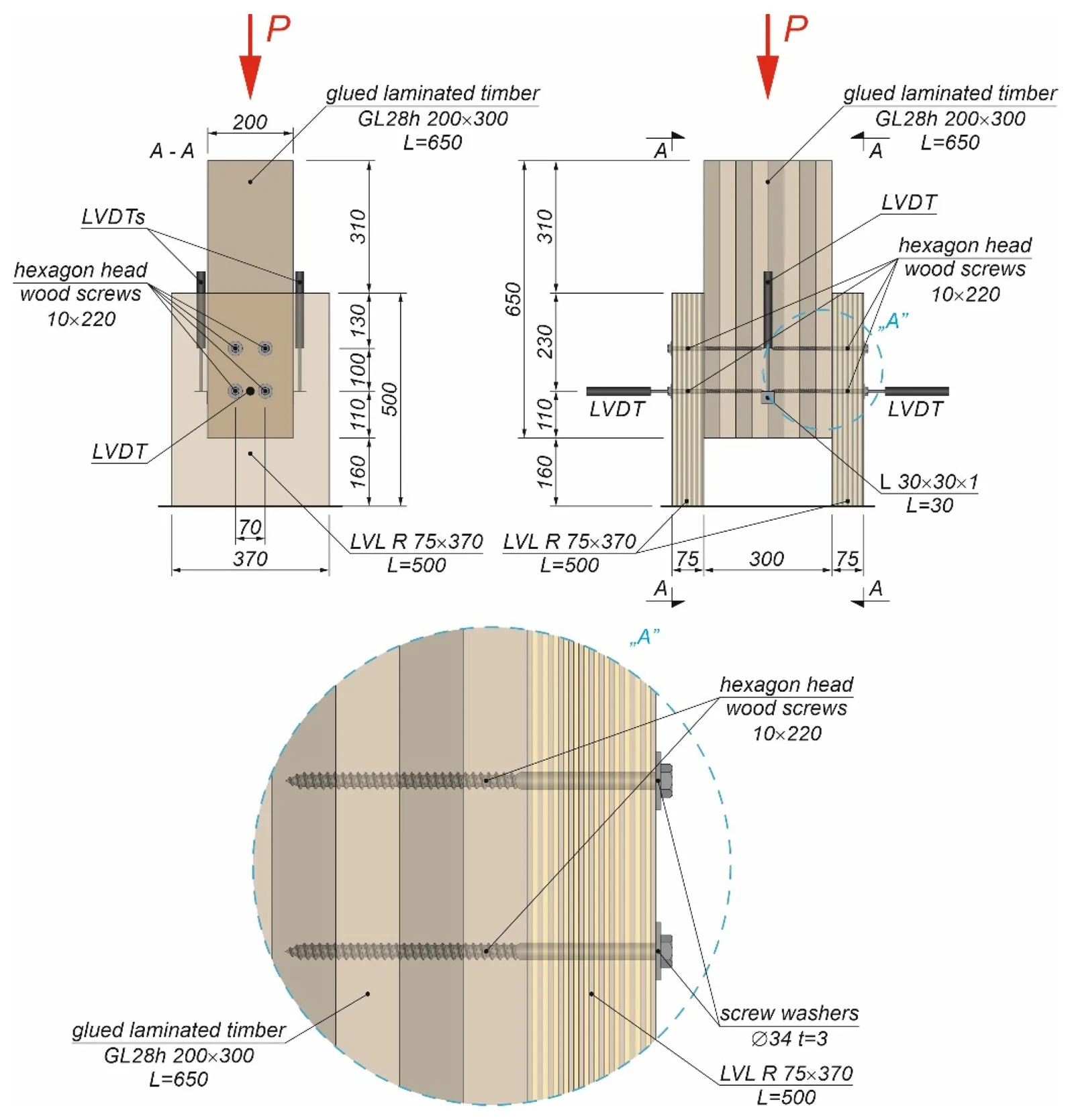
Location of LVDTs on the specimen with 10 × 220 mm hexagon-head wood screws and 75 mm LVL R slabs.

**Figure 4:**
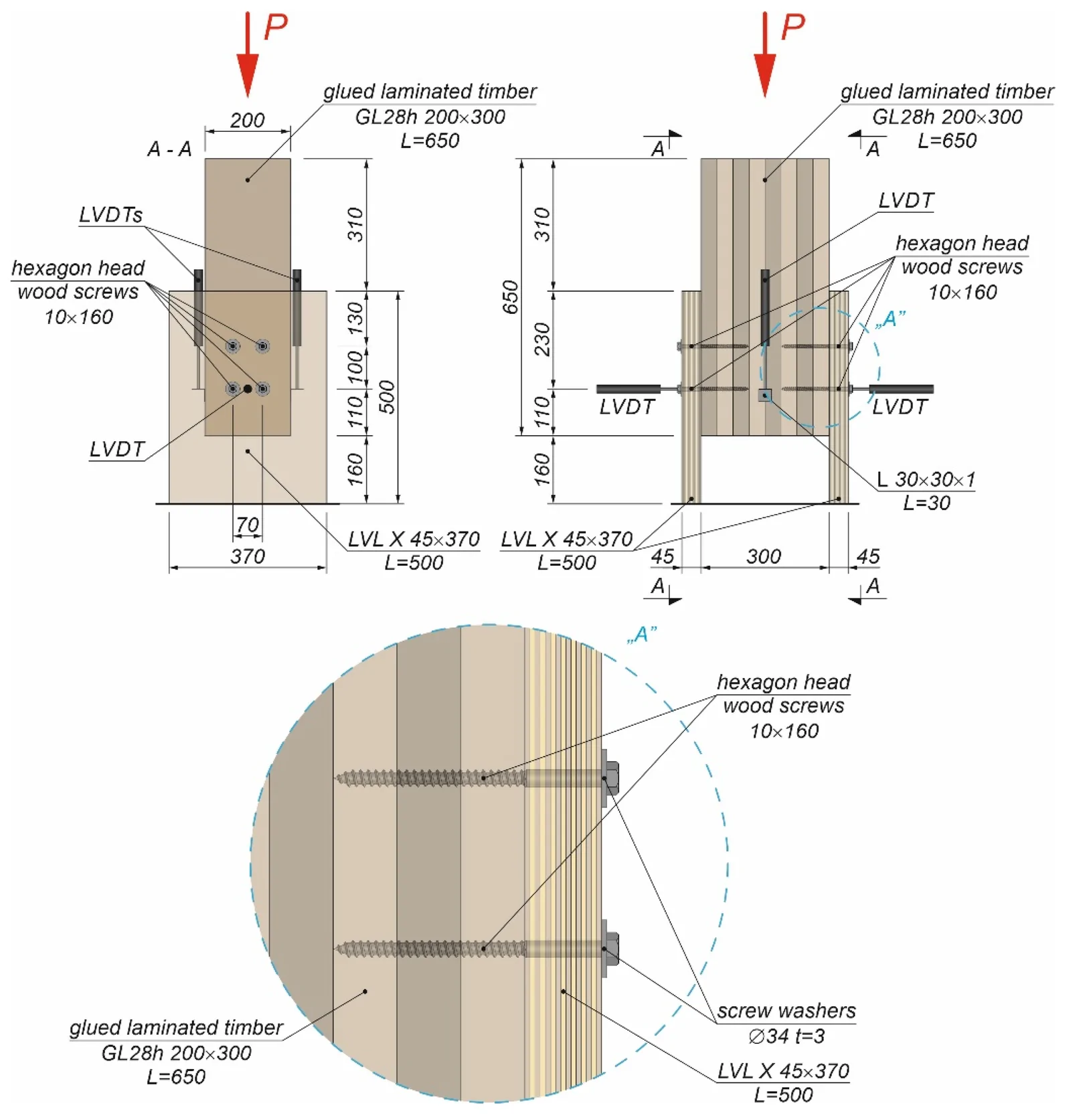
Location of LVDTs on the specimen with 10 × 160 mm hexagon-head wood screws and 45 mm LVL X slabs.

**Figure 5:**
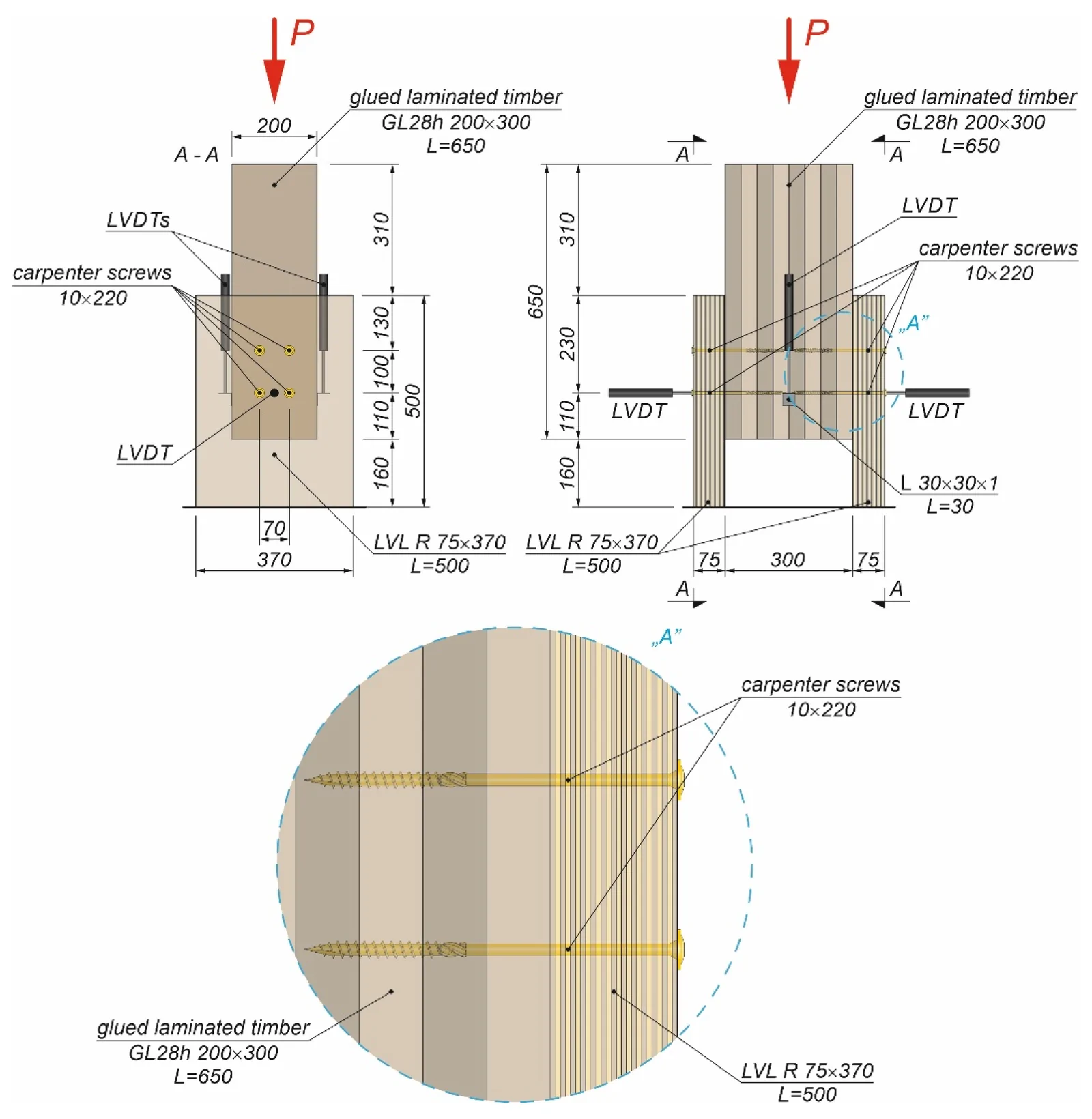
Location of LVDTs on the specimen with 10 × 220 mm carpenter screws and 75 mm LVL R slabs.

**Figure 6:**
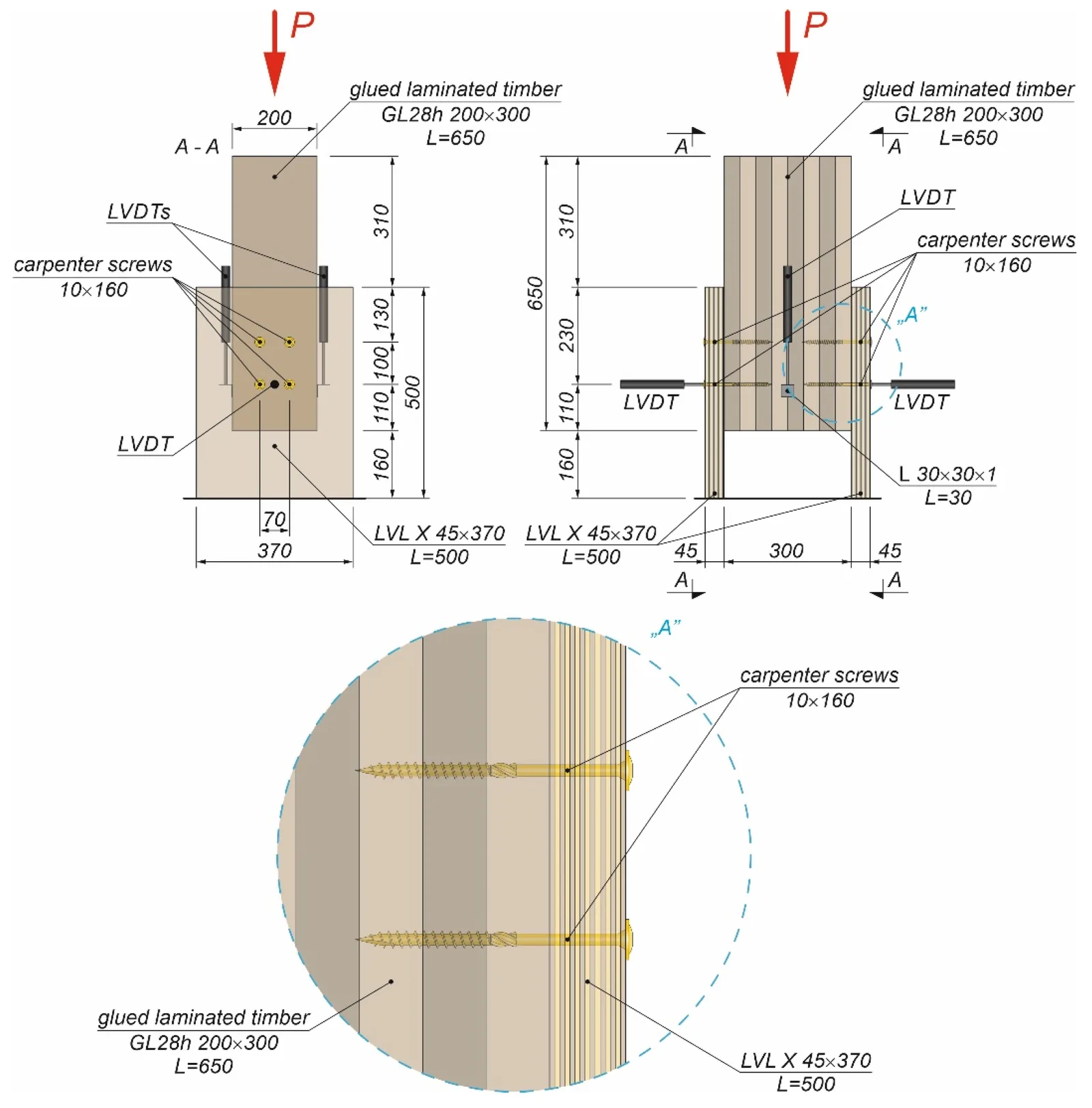
Location of LVDTs on the specimen with 10 × 160 mm carpenter screws and 45 mm LVL X slabs.

The laboratory tests were conducted on symmetrical specimens consisting of two LVL slabs and a GLT beam (Figure 2, Figure 3, Figure 4, Figure 5 and Figure 6). The use of symmetrical specimens ensured a uniform load distribution [23]. Four groups of specimens varying in screw type and slab thickness were used (Table 3). The vertical LVDTs measured the relative displacement of the GLT beam with respect to the supported LVL slabs, which was taken as the connection slip. No noticeable vertical displacement or indentation of the LVL slabs at the rigid steel supports was observed during the tests. Furthermore, two LVDTs were used to measure the horizontal movement of the LVL slabs. The specimens were loaded using an Instron 8505 Plus machine (Instron, HighWycombe, UK). The horizontal displacement of the LVL slabs was monitored as an auxiliary control measurement to identify any possible lateral movement or instability of the specimens. The recorded horizontal displacements remained small compared with the vertical connection slip, and no systematic lateral movement affecting the load–slip response was observed. Therefore, these measurements were not used in the subsequent evaluation of the mechanical properties of the connections.

For the C_10_45, H_10_45, and C_10_75 configurations, the predrilled-hole diameter was 7 mm. During preliminary assembly of the H_10_75 configuration, one 10 × 220 mm hexagon-head screw fractured during insertion into a 7 mm hole. Consequently, a 9 mm through-hole was adopted in the LVL for all subsequently tested H_10_75 specimens. The predrilled-hole depths were 100 mm for C_10_45 and H_10_45, 130 mm for C_10_75, and 145 mm for H_10_75. The screws were installed using a torque wrench (Sandvik Belzer, IZO-I-100, 10–100 Nm) with specified insertion torque levels of 25 Nm for carpenter screws, 50 Nm for 10 × 160 mm hexagon-head screws, and 47.5 Nm for 10 × 220 mm hexagon-head screws. Individual torque values for each screw were not recorded. The spaces between the screws were 70 mm in the transverse direction and 100 mm in the longitudinal direction. Experimental tests were conducted in accordance with [24]. In the first stage, a load control regime was used to obtain a regular shape of the force–slip curve. First, the shear load was increased from 0 to 40% of the estimated ultimate load over two minutes and it remained at this level for half a minute. Subsequently, the load was decreased from 40% to 10% of the estimated ultimate load and maintained at this level for an additional half a minute. Next, the load was increased from 10% to 70% of the estimated ultimate load. From that moment on, the push-out test was conducted using a displacement control regime with a piston velocity of 5.0 mm/min. The initial loading levels were estimated with reference to the analytical load-carrying capacity obtained from Equation (1) and the number of screws in one specimen (8). During the experimental programme, the loading levels were subsequently adjusted on the basis of the preceding test results. The formula for the calculation of the load-carrying capacity of the screwed connection (per one connector) developed by Hassanieh et al. [25] is presented below:

(1) $$P_{pred}\;=\;(5.95d\;-\;27.2)/2,$$

where *P_pred_* is the predicted load per one connector, *d* is the screw diameter, the coefficient 5.95 has units of kN/mm, and the constant 27.2 has units of kN. The division by two expresses the original relation on a per-connector basis.

The estimated ultimate load and the loading procedure were modified during the tests taking into account the previous results. In Equation (1), *d* denotes the nominal screw diameter and should not be confused with the diameter of the predrilled hole. Both screw types investigated in this study had a nominal diameter of 10 mm. Equation (1) therefore gives the same reference analytical capacity for both connector types. However, the formulation does not explicitly distinguish between core/shank diameter, thread diameter or thread geometry.

Although Equation (1) was originally developed for LVL-to-steel connections, it was adopted in the present study only as a reference analytical formulation for benchmarking the capacity of the investigated TTC connections. Its application is motivated by similarities in the local LVL–connector interaction, particularly timber embedment and local deformation around the fastener. Nevertheless, the GLT component introduces additional deformation mechanisms that are not represented in the original LVL-to-steel formulation. Therefore, Equation (1) is not treated here as a validated design model for TTC connections.

For the evaluation of connection stiffness, two secant stiffness parameters were considered. The initial stiffness *k_pre_* was determined on the first loading branch, immediately before the prescribed unloading cycle, as

(2) $$k_{pre}=\frac{F_{pre}/8}{s_{pre}},$$

where *F_pre_* and *s_pre_* are the specimen load and corresponding slip immediately before unloading. The post-cycle secant stiffness *k*_0.6_ was determined at a load level equal to 0.6*F_peak_* on the reloading branch following the loading–unloading cycle:

(3) $$k_{0.6}\;=\;\frac{0.6F_{ult}/8}{s_{0.6}}.$$

Here, *F_ult_* denotes the experimentally measured peak load of the specimen and *s*_0.6_ is the corresponding slip on the reloading branch. Since each specimen contained eight screws, the reported force and stiffness values were expressed as equivalent average values per connector by dividing the total specimen load by eight. Individual connector forces were not measured.

## 3. Results and Discussion

**Figure 7:**
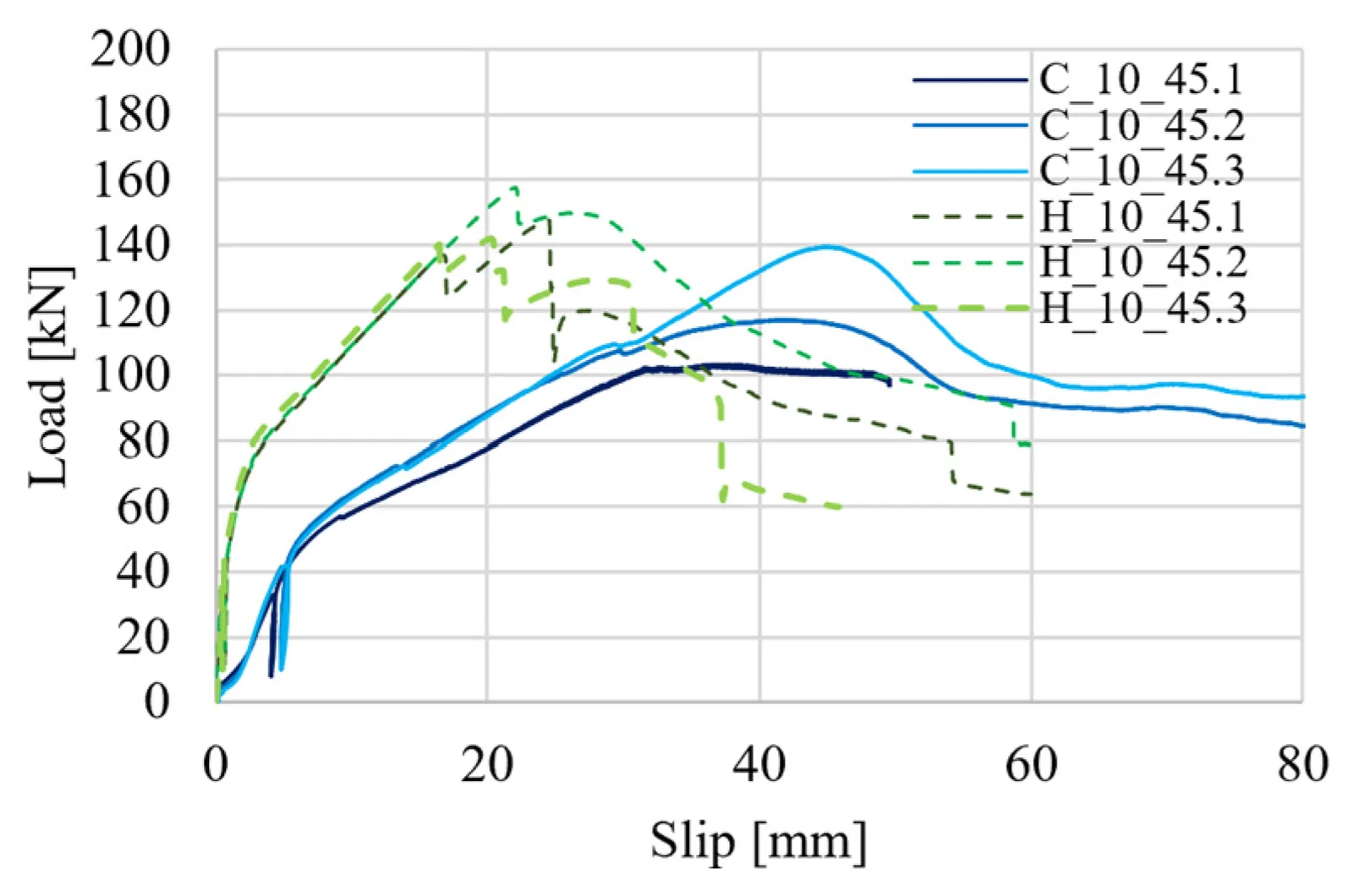
The load–slip curves from the push-out tests of the specimens using 45 mm LVL X slabs.

**Figure 8:**
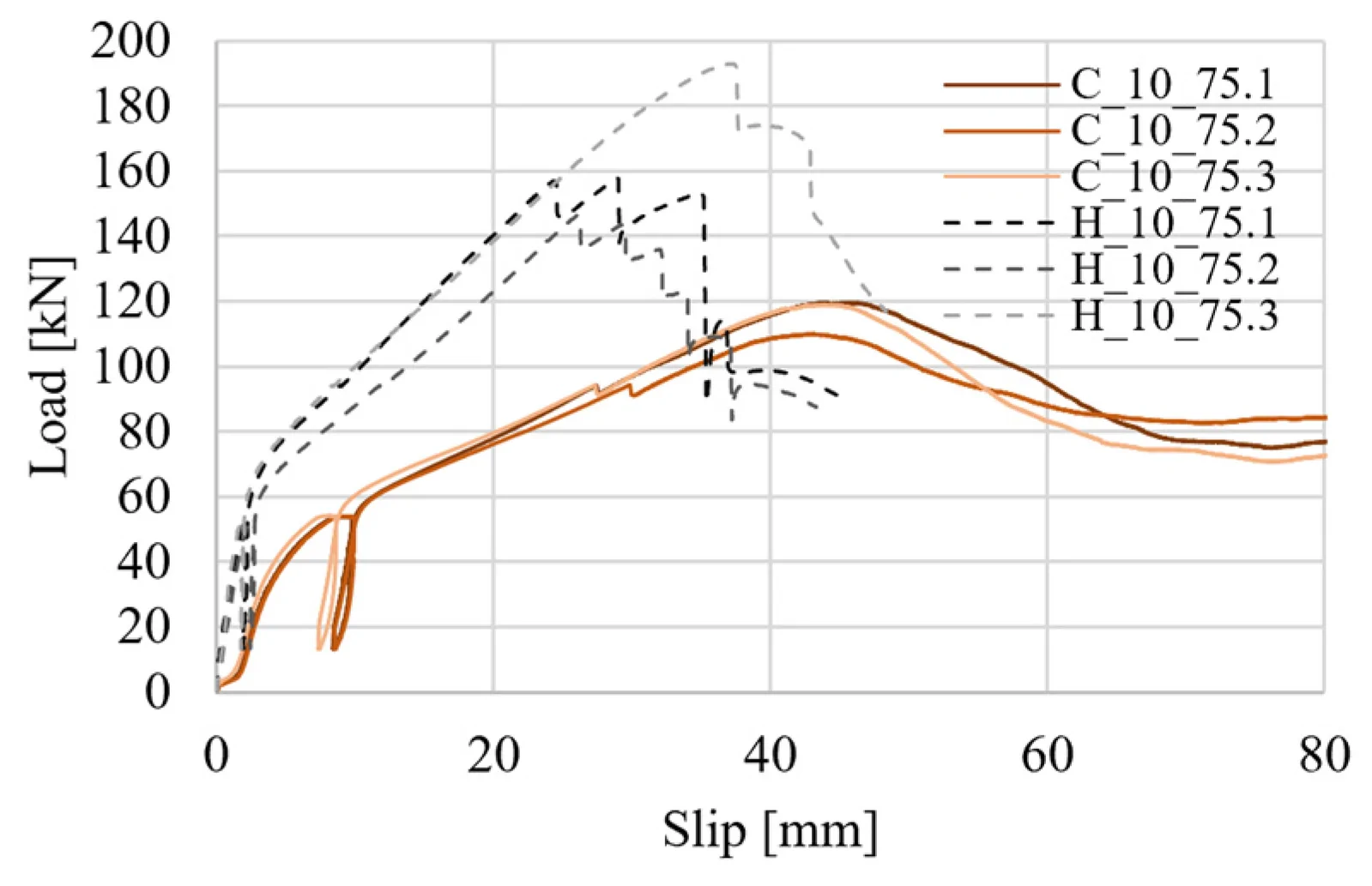
The load–slip curves from the push-out tests of the specimens using 75 mm LVL R slabs.

The results of the push-out tests are presented in Figure 7 and Figure 8, and in Table 4, Table 5, Table 6 and Table 7. The following symbols are used in Table 4, Table 5, Table 6 and Table 7: *P_ult_*—equivalent peak load per connector; *s_ult_*—slip corresponding to the experimentally measured peak load; *k_pre_*—initial secant stiffness determined immediately before the unloading cycle; and *k*_0.6_—secant stiffness determined at 0.6*F_ult_* on the subsequent reloading branch.

**Figure 9:**
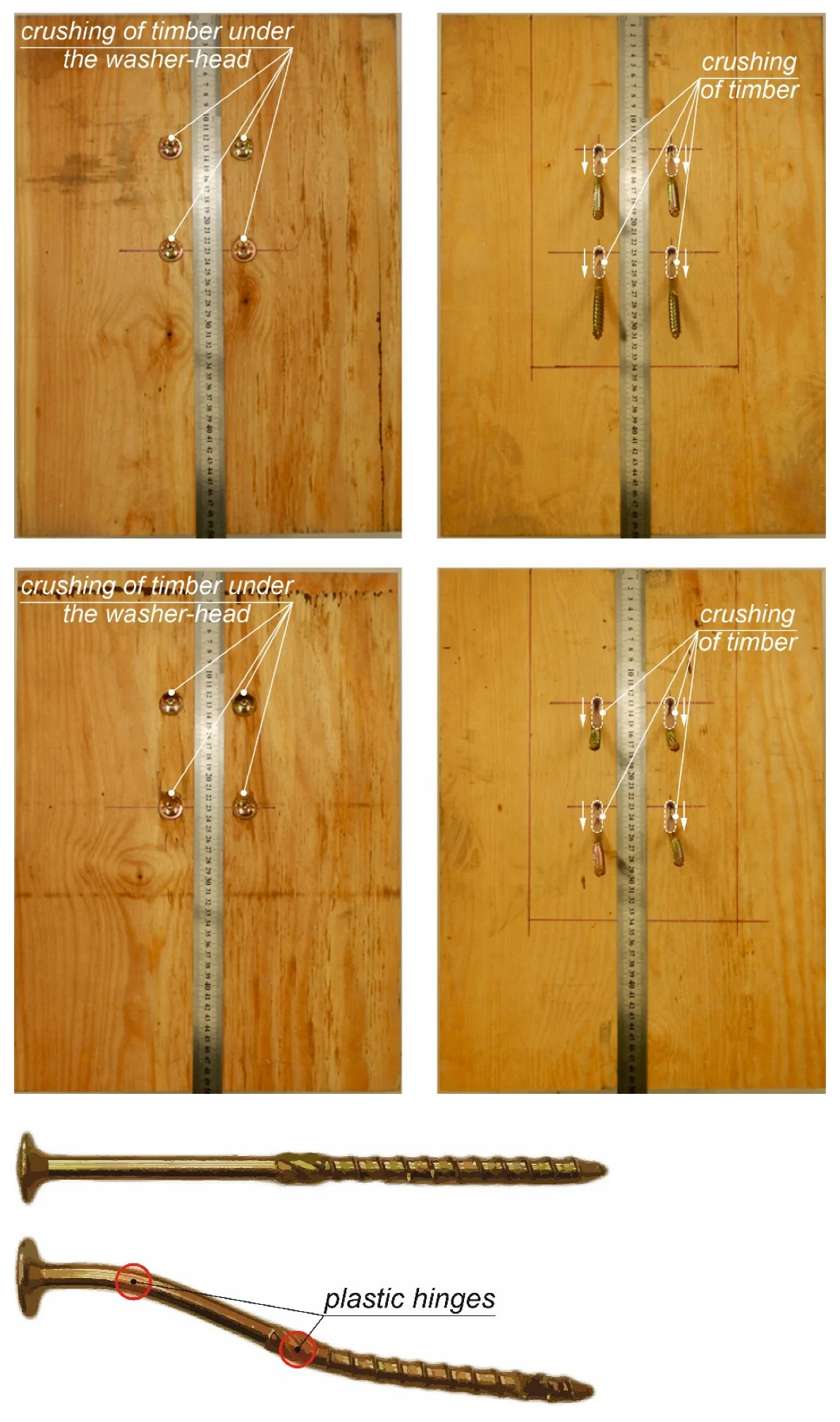
The mode of failure of the shear connections with carpenter screws 10 × 160 and LVL X 45 (C_10_45) and damaged screws after push-out test, arrows represent direction of the GLT beam movement.

**Figure 10:**
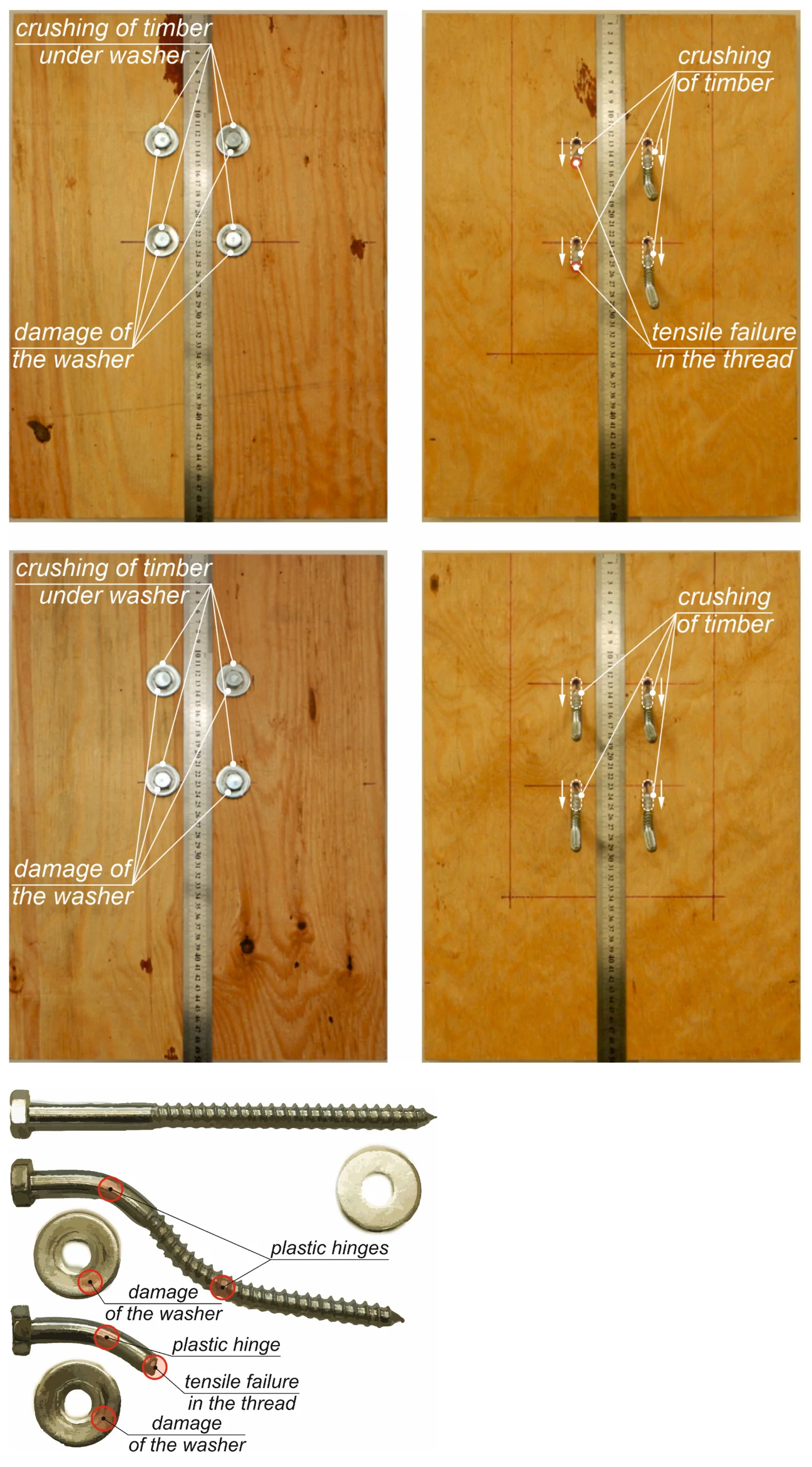
The mode of failure of the shear connections using 10 × 160 mm hexagon-head wood screws and 45 mm LVL X slabs (H_10_45), arrows represent direction of the GLT beam movement.

**Figure 11:**
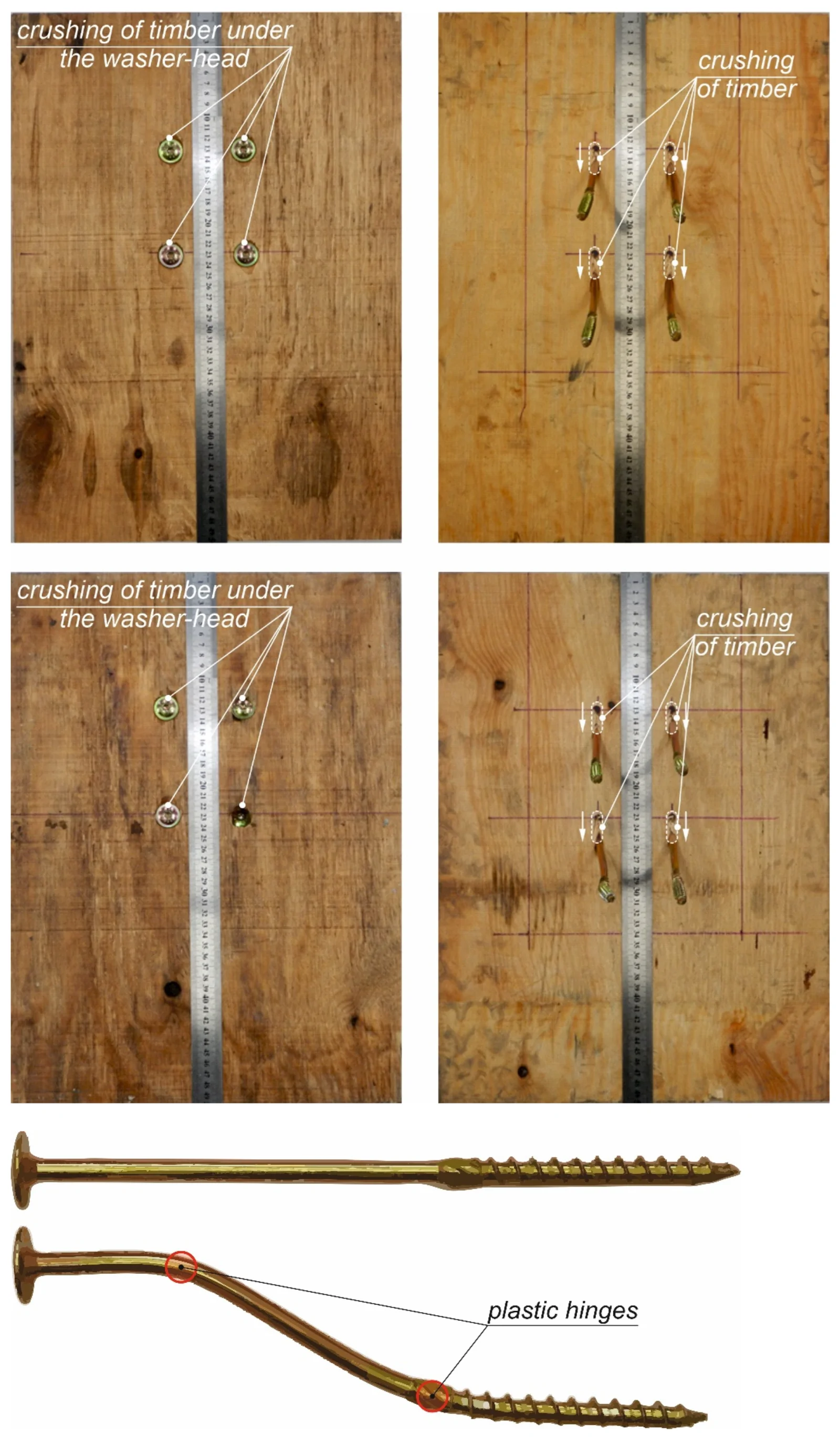
The mode of failure of the shear connections with 10 × 220 mm carpenter screws and 75 mm LVL R slabs (C_10_75), arrows represent direction of the GLT beam movement.

**Figure 12:**
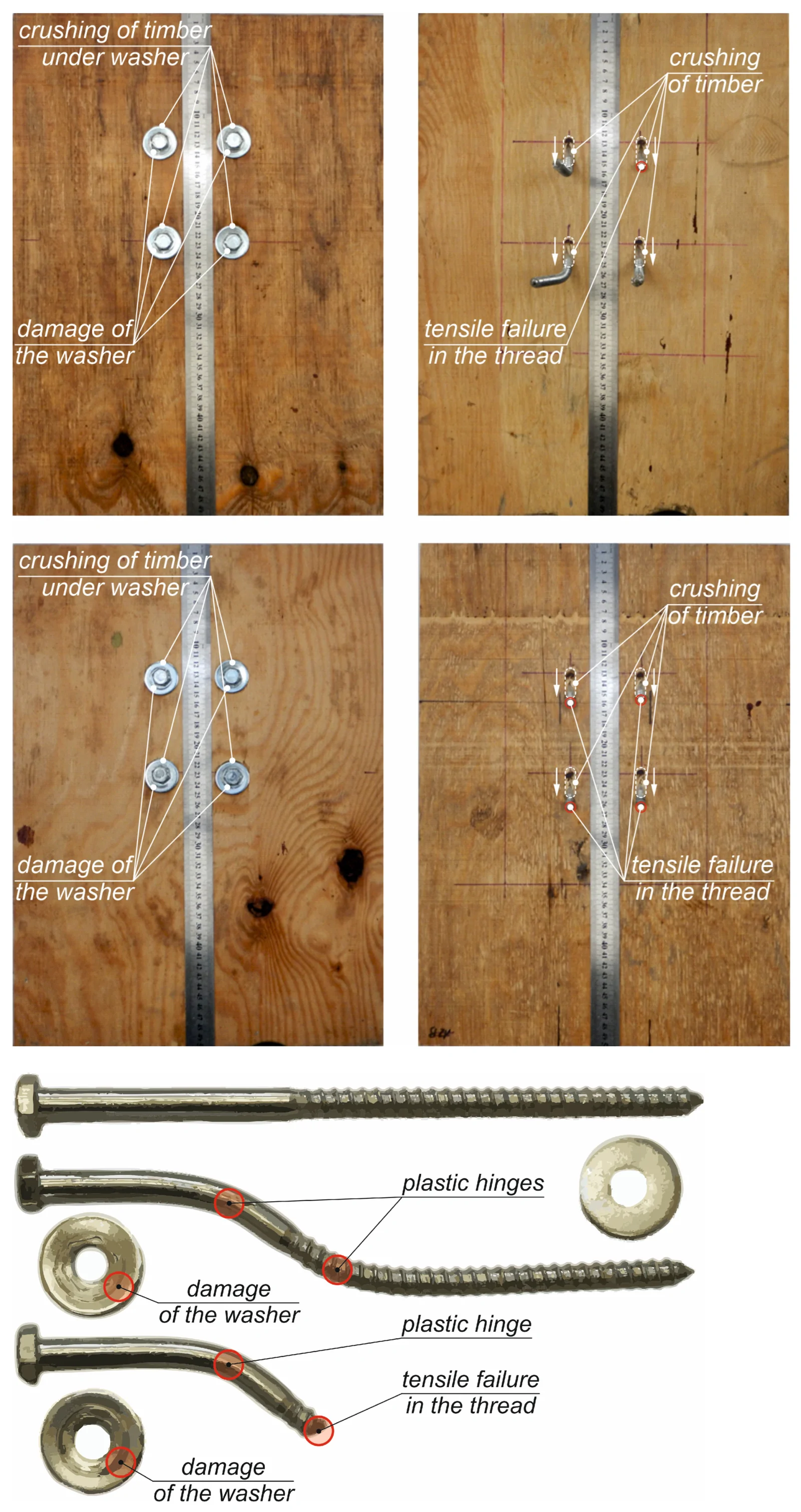
The mode of failure of the shear connections with hexagon 10 × 220 mm head wood screws and 75 mm LVL R slabs (H_10_75), arrows represent direction of the GLT beam movement.

The connections using carpenter screws had a higher slip corresponding to the ultimate load per one connector than the connections using hexagon-head wood screws. The mean slip corresponding to *P_ult_* of the C_10_45 specimens was 1.9 times higher than the mean *s_ult_* of the H_10_45 specimens. The mean slip corresponding to *P_ult_* of the C_10_75 specimens was 1.4 times higher than the mean *s_ult_* of the H_10_75 specimens. Within the configurations tested, the connections with carpenter screws exhibited greater ultimate slip than those with hexagon-head wood screws, indicating a more ductile response. However, the connections using hexagon-head wood screws showed higher stiffness and load-carrying capacity than the connections with carpenter screws. For the tested configurations, the H-series exhibited higher initial secant stiffness *k_pre_* than the corresponding C-series. A reduction in secant stiffness was observed after the prescribed loading–unloading cycle, as reflected by the lower *k*_0.6_ values. This difference between *k_pre_* and *k*_0.6_ illustrates the progressive nonlinear response of the connections with increasing load and accumulated slip. The ultimate load per one connector of the H_10_45 specimens was 1.3 times higher than the one of the C_10_45 specimens. The ultimate load per one connector of the H_10_75 specimens was 1.4 times higher than the one of the C_10_75 specimens. The tested connections were ductile. Each connection exhibited a characteristic slip capacity greater than 6 mm, recommended by Eurocode 4 [26]. The failure modes of the specimens are presented in Figure 9, Figure 10, Figure 11 and Figure 12. The authors observed two distinctive modes of failure (C and H). The failure mode depended on the screw type. Mode C was observed in the specimens with carpenter screws. It was associated with the formation of two plastic hinges within every screw, and the local crushing of the timber material in the LVL and GLT members. Mode H was observed in the specimens using hexagon-head wood screws. This mode was associated with the formation of two plastic hinges within a single screw, the local crushing of the timber material in the LVL and GLT members, and the tensile failure in the threaded region of some screws.

According to Table 8, Equation (1) gives a reference analytical capacity of 16.2 kN per connector for all investigated configurations because both screw types have the same nominal diameter of 10 mm. The experimental-to-predicted ratios were 0.88 and 0.90 for the C_10_45 and C_10_75 configurations, respectively, and 1.15 and 1.28 for the H_10_45 and H_10_75 configurations. Thus, Equation (1) slightly overpredicted the capacity of the tested carpenter screw configurations and underpredicted that of the tested hexagon-head screw configurations.

The identical analytical prediction for both screw types illustrate one limitation of Equation (1): the formulation does not distinguish between core/shank diameter, thread diameter or thread geometry. These differences may contribute to the observed discrepancies between the analytical and experimental capacities. Moreover, deformation of the GLT member and the LVL–GLT interaction are not explicitly represented in the original LVL-to-steel formulation.

## 4. Numerical Approach

### 4.1. Constitutive Modelling and Numerical Assumptions

**Figure 13:**
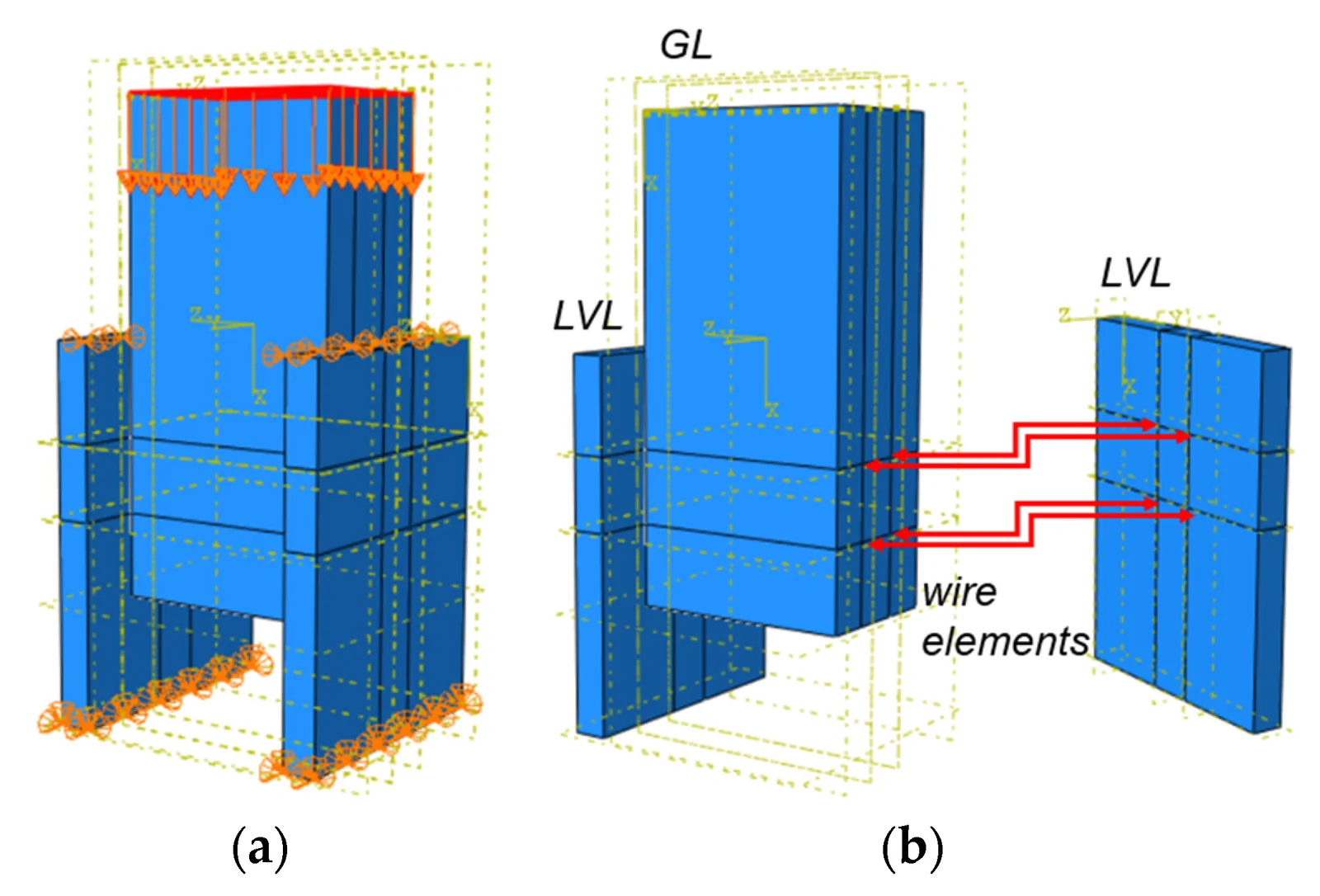
Visualisation of the FE model in Abaqus: (**a**) boundary conditions (translational constraint at the LVL base and horizontal restraint at the upper edge) and displacement-controlled loading at the top of the GLT; (**b**) wire elements between the GLT and LVL.

The numerical models were developed in Abaqus (v2025) (Figure 13a) primarily to assess whether the experimentally derived tri-linear load–slip relationships could be consistently represented by discrete spring connectors and subsequently used in more complex TTC structural models. The push-out simulations therefore served as a verification and calibration step rather than as a detailed simulation of the local screw–timber interaction. In particular, the orthotropic-plastic material model adopted for the LVL and GLT components was intended to enable subsequent analyses of full-scale TTC members, where material nonlinearity may become significant.

The LVL and GLT members were discretised using C3D8R solid elements with an average mesh size of approximately 20 mm. The shear connectors were represented by nonlinear spring elements whose mechanical characteristics were derived directly from the experimental load–slip response (Figure 13b). This modelling strategy enables the global behaviour of the connections to be reproduced with substantially lower computational effort than a detailed local model of the screw–timber interaction.

Independent material-level shear tests of the screws were not performed; consequently, shear strength, ultimate shear deformation, and shear modulus are not reported for the tested fasteners. These parameters were not required as direct FE input because the connector response was represented by experimentally calibrated nonlinear spring characteristics.

**Figure 14:**
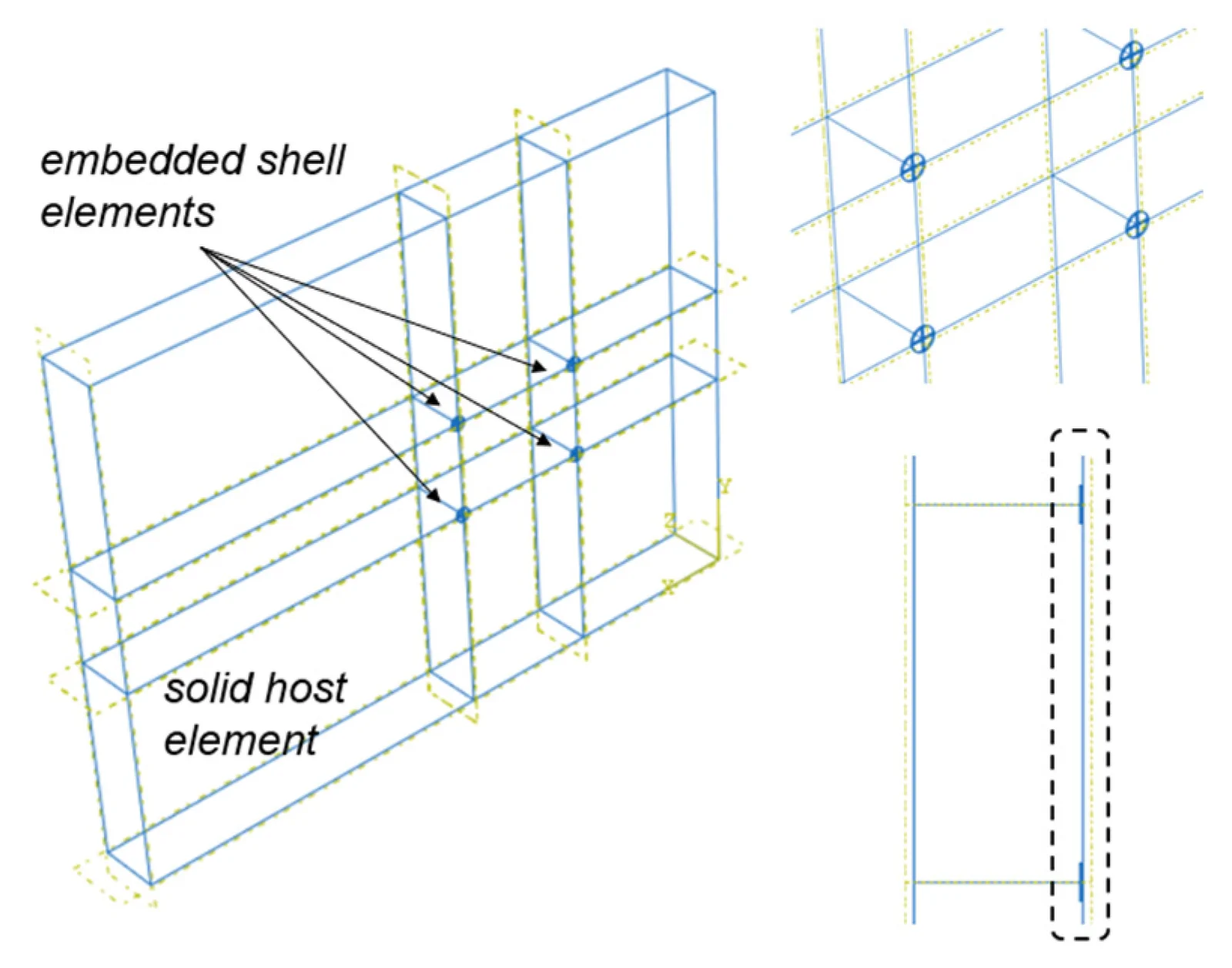
Model representation of embedded shell elements within a 45 mm LVL X solid host element.

To facilitate the transfer of forces between the discrete spring connectors and the C3D8R solid elements, thin shell patches were introduced and embedded in the corresponding LVL and GLT host regions (Figure 14). The embedded-element technique is a standard modelling functionality available in Abaqus and was used here as a numerical coupling approach rather than as a physical representation of the screw–timber contact surface. Each shell patch was assigned the same material properties as the corresponding host material and was fully embedded in the surrounding solid mesh. The centres of the opposing shell patches were connected by the nonlinear spring element representing the mechanical behaviour of the connector. In this way, the connector force was transferred to the surrounding solid region instead of being applied directly to a single solid node, reducing the sensitivity of the model to local single-node force transfer. The embedded shell patches were circular with a diameter of 20 mm, approximately corresponding to the local solid-mesh discretisation. The load was applied as a prescribed displacement imposed on the top surface of the GLT element. Friction between the LVL and GLT contact surfaces was not modelled explicitly. Its contribution to the global connection response is implicitly included in the experimentally calibrated nonlinear spring characteristic.

In the initial modelling approach, an advanced constitutive material model was adopted, accounting for both orthotropy (O) and plasticity (Pl). Material failure was described using the six-parameter Hill yield criterion. The material properties implemented in the numerical models of the structural elements are summarised in Table 9. It should be noted that the engineering constants adopted in the FE material models are not the nominal material properties reported in Table 1 and Table 2. The longitudinal elastic moduli of LVL X and LVL R were adopted from the experimentally identified orthotropic material models reported in [27], whereas the GLT engineering constants were adopted from the literature-based orthotropic model reported in [28,29]. These parameters were not calibrated against the push-out tests investigated in the present study.

The Hill parameters (see Table 10) were calculated based on the equations presented in [28,29,30,31]. For the orthotropic material model, the material coordinate system was defined so that direction 1 corresponds to the longitudinal axis of the elements (i.e., the largest dimension, aligned with the direction of the applied load), direction 2 represents the specimen width, and direction 3 corresponds to the specimen thickness.

The Hill criterion extends the von Mises yield condition to anisotropic materials by introducing six independent parameters (*R*11, *R*22, *R*33, *R*12, *R*13, and *R*23), which weigh the contributions of the normal and shear stress components and enable different yielding responses along the principal material directions [32]. These parameters are typically calibrated based on experimental yield stresses measured in different loading directions and are widely used to model anisotropic plasticity in metallic and composite materials.

**Figure 15:**
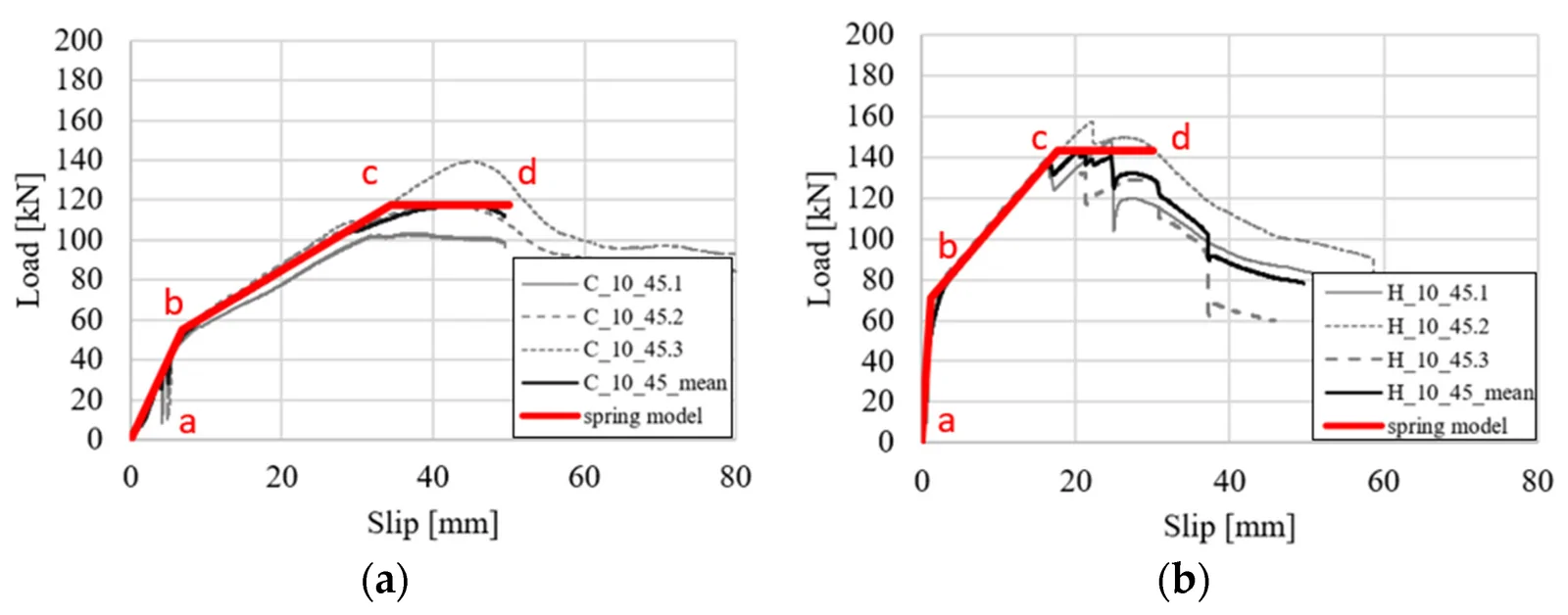
Tri-linear fit of load–slip curves from push-out tests on specimens with 45 mm LVL X slabs: (**a**) carpenter screws; (**b**) hexagon-head wood screws; a, b, c and d—points of a tri-linear load–slip response.

**Figure 16:**
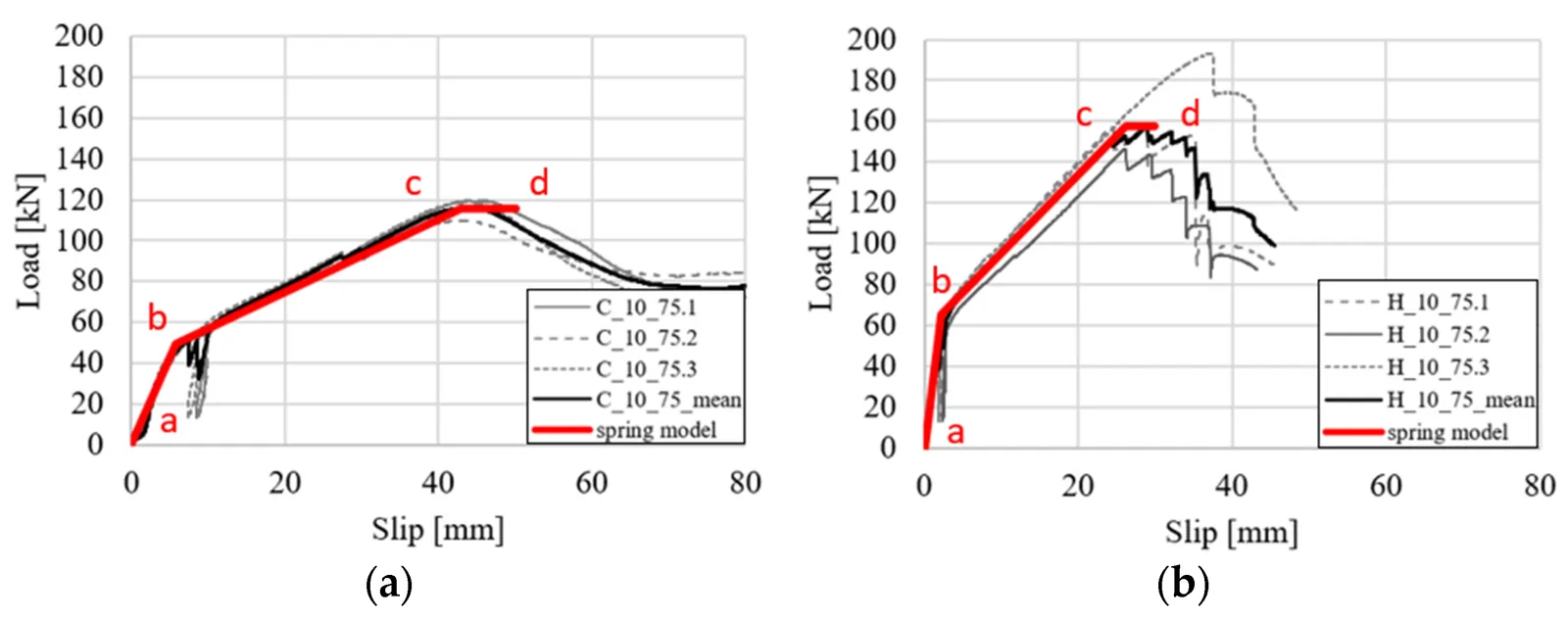
Tri-linear fit of load–slip curves from push-out tests on specimens with 75 mm LVL R slabs: (**a**) carpenter screws; (**b**) hexagon-head wood screws; a, b, c and d—points of a tri-linear load–slip response.

Based on the obtained load–slip curves (Figure 7 and Figure 8), an average response was derived (Figure 15 and Figure 16). It was subsequently used to define the load–slip characteristics for the four analysed cases, which were adopted as the constitutive relationships of the springs and represented by a tri-linear model (red line). Since each push-out test represented a shear connection composed of multiple connectors, the resulting characteristic curve was subsequently scaled to obtain the response of a single connector.

Numerically, the connector was modelled using a spring with a tri-linear load–slip response (segments ab–bc–cd), which reflected the deformation mechanism observed in the experimental tests. The first segment (ab) corresponded to the elastic behaviour of the connector. The second segment (bc) captured the reduction in stiffness associated with the indentation of the connector into the LVL element and the formation of the first plastic hinge. The third segment (cd) corresponded to the development of the second plastic hinge, which led to a flattening of the equilibrium path. The stiffness values adopted for individual connectors are summarised in Table 11.

For each connection configuration, a mean experimental load–slip response was first obtained from the three tested specimens. The tri-linear approximation was then derived from this mean curve. Three response ranges were identified: (ab) the initial high-stiffness response, (bc) the subsequent reduced-stiffness response, and (cd) the post-peak plateau. Linear functions were fitted to the (ab) and (bc) ranges using spreadsheet-based tools, and their intersection defined point b. Point c corresponded to the transition to the post-peak plateau, while segment cd was represented by zero tangent stiffness. The procedure was applied consistently to all four tested configurations. The tri-linear model should therefore be interpreted as a simplified deterministic representation of the mean experimental response rather than as a statistical model of the connector population.

Additionally, it should be emphasised that, in the presented numerical model, the fasteners were not modelled by means of a detailed wood foundation model. For example the model proposed by Hong et al. [33,34] is a more advanced approach that enables the local reduction in the timber foundation around the connectors to be taken into account and is particularly useful when the local embedment behaviour and stress redistribution around fasteners are analysed explicitly. In this study, however, the objective of the FEM model was to reproduce the global load–slip response of the tested TTC connections rather than to describe the local embedment mechanism around each connector. For this reason, the connectors were represented by discrete connector elements calibrated directly on the basis of the experimental load–slip curves. This modelling strategy reduces the risk of overestimating the global stiffness of the connection, since the local effects associated with timber embedment, fastener rotation, local crushing and progressive slip are included implicitly in the experimentally calibrated force–slip law. Nevertheless, it should be noted that the adopted connector model does not provide a detailed description of the local stress state in the timber surrounding the screws. Consequently, the model should be interpreted as a calibrated global model applicable to the investigated connection configurations, and not as a general predictive model for arbitrary screw arrangements or timber foundation conditions. A more detailed modelling approach based on a wood foundation model, such as that proposed by Hong et al., should be considered in future work when the local embedment behaviour of STSs is investigated explicitly.

Although the elastic material model was sufficient to reproduce the global response of the push-out specimens, the orthotropic-plastic Hill model was retained to establish a modelling framework suitable for subsequent analyses of more complex TTC members, such as four-point bending simulations, where nonlinear behaviour of the LVL and GLT components may become relevant.

### 4.2. Results of Numerical Simulations

**Figure 17:**
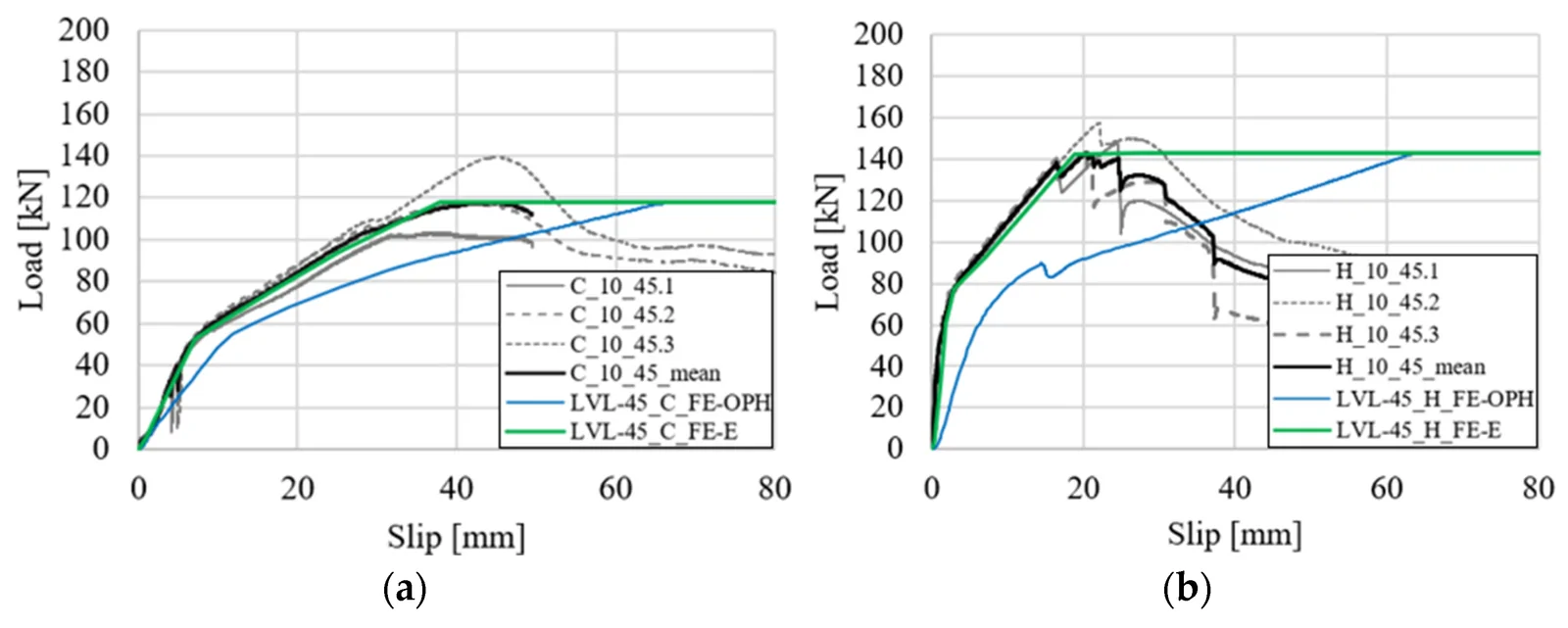
Comparison of numerical load–slip curves obtained using the orthotropic plastic material model with the Hill yield criterion (OPH) and the elastic (E) material model with experimental curves from push-out tests on specimens with 45 mm LVL X slabs: (**a**) carpenter screws; (**b**) hexagon-head wood screws.

**Figure 18:**
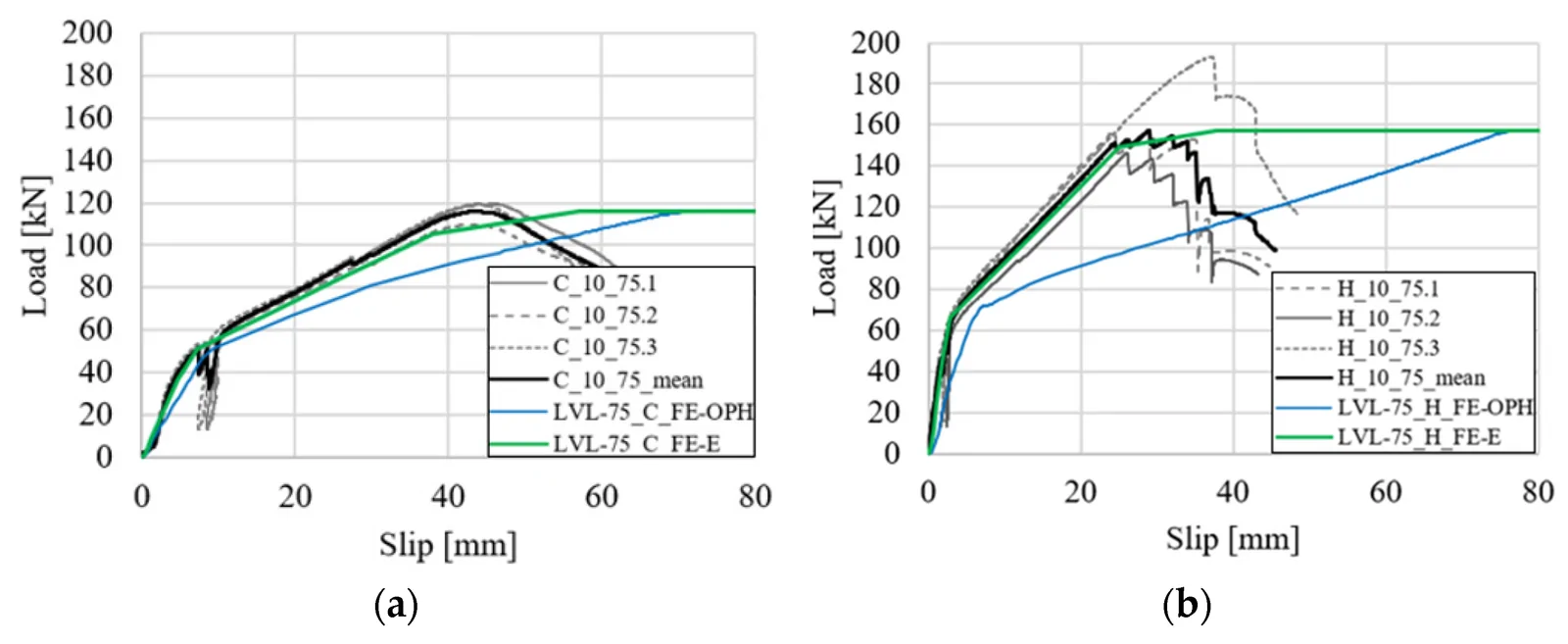
Comparison of numerical load–slip curves obtained using the orthotropic plastic material model with the Hill yield criterion (OPH) and the elastic (E) material model with experimental curves from push-out tests on specimens with 75 mm LVL R slabs: (**a**) carpenter screws; (**b**) hexagon-head wood screws.

As stated previously, the initial modelling strategy employed a complex constitutive material model for LVL and GLT, incorporating orthotropy, plasticity, and the Hill yield criterion (OPH). For the spring properties and constitutive parameters defined in Table 11, the resulting numerical equilibrium paths were consistently underestimated relative to the mean experimental response (see Figure 17 and Figure 18, solid blue lines). This discrepancy indicates that the plastic deformation effects in the timber elements were accounted for twice: explicitly through the nonlinear constitutive material formulation and implicitly through the load–slip characteristics of the connector springs derived from the laboratory tests. To eliminate this redundancy, subsequent analyses were conducted using a purely elastic isotropic (E) material model. The corresponding numerical responses (green curves in Figure 17 and Figure 18) were in close agreement with the experimental results.

**Figure 19:**
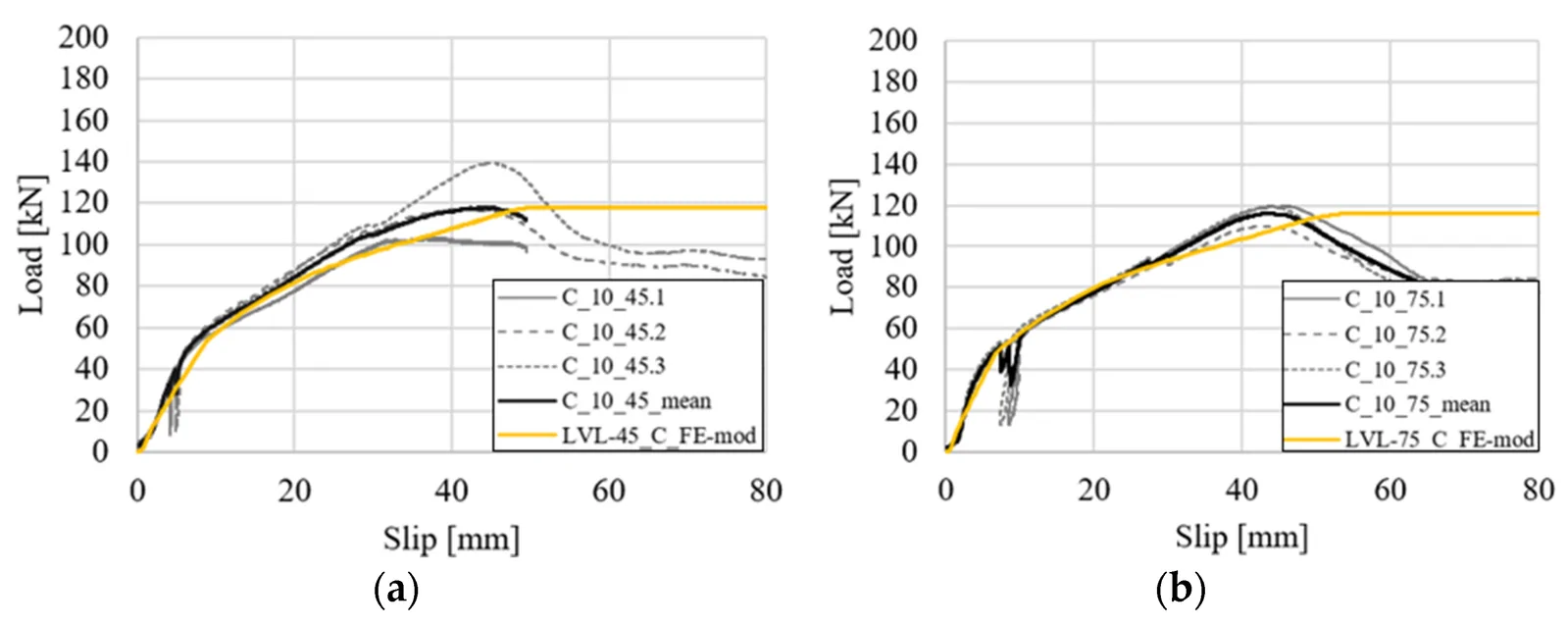
Comparison of numerical load–slip curves obtained using the orthotropic plastic material model with the Hill yield criterion (OPH) and modified spring stiffness according to Table 12 with experimental curves from push-out tests on specimens with carpenter screws and: (**a**) 45 mm LVL X slabs; (**b**) 75 mm LVL R slabs.

However, in the numerical modelling of composite beams, plasticity effects in the timber components cannot be neglected. At the same time, simplified connector modelling based on experimentally obtained shear load–slip relationships is desirable. To reconcile these requirements, the spring characteristics presented in Figure 15 and Figure 16 were rescaled by stiffness correction factors *β*_ab_ and *β*_bc_, applied to segments (ab) and (bc) of the tri-linear spring model, respectively. The resulting correction factors for the analysed specimens are summarised in Table 12. The numerical response obtained using the modified spring stiffness in combination with the full constitutive material model (orthotropy, plasticity, and Hill yield criterion, OPH) is represented by orange line in Figure 19. The agreement with the experimental data is satisfactory, indicating that the proposed modelling strategy provides a consistent representation of both material nonlinearity and connector behaviour.

## 5. Conclusions

This paper investigated the mechanical behaviour of TTC connections using two types of mechanical fasteners: hexagon-head wood screws and carpenter screws. For the configurations investigated in this study, connections employing hexagon-head wood screws exhibited higher stiffness and load-carrying capacity, whereas the configurations with carpenter screws developed larger slip values at ultimate load per connector. These differences should not be attributed solely to the screw type, since the tested configurations also differed in selected geometrical and installation parameters. Therefore, the observed differences in stiffness, load-carrying capacity, and ductility are restricted to the specific connection configurations investigated in this study.

Both connection types demonstrated a characteristic slip capacity exceeding the recommended threshold for ductile connections (6 mm). In all specimens, failure mechanisms were governed by the formation of two plastic hinges in the screws combined with local crushing of the timber material in the LVL and GLT members. For connections with hexagon-head wood screws, additional tensile failure in the threaded region of some screws was observed, resulting in lower overall ductility compared to carpenter screw connections.

The analytical model proposed by Hassanieh et al. for estimating the load-carrying capacity of STC screwed connections was applied to the investigated TTC joints. The analytical formulation proposed by Hassanieh et al. was used as a reference benchmark for the investigated TTC connections. Experimental-to-predicted capacity ratios ranged from 0.88 to 0.90 for the tested carpenter screw configurations and from 1.15 to 1.28 for the tested hexagon-head screw configurations. The observed differences highlight the limitations of applying a formulation based primarily on nominal screw diameter to connectors with different core/shank and thread geometries. In addition, non-uniform load sharing among the fasteners may have contributed to the discrepancy between the analytical and experimental capacities. In particular, the stepwise load drops observed for some hexagon-head screw specimens suggest progressive connector failure and subsequent redistribution of forces between the remaining fasteners. Since individual connector forces were not measured, this interpretation should be regarded as qualitative. As the original model was developed for LVL-to-steel connections, its application to the present TTC joints should therefore be regarded as comparative rather than predictive.

The experimental load–slip relationships obtained in this study enable the determination of the number of connectors required to achieve a desired degree of composite action in TTC beams. Moreover, these load–slip curves provide a reliable basis for numerical simulations of composite beams employing either hexagon-head wood screws or carpenter screws as shear connectors.

The numerical analyses demonstrated that the direct combination of an orthotropic plastic timber material model with the Hill yield criterion (OPH) and experimentally derived nonlinear connector characteristics leads to a systematic underestimation of the structural response due to double accounting of plastic deformation effects. While adopting a purely elastic isotropic material model eliminates this redundancy and yields good agreement with experimental results, such simplification is not appropriate for the analysis of composite beams where material plasticity must be explicitly considered.

To address this issue, a stiffness rescaling procedure for the tri-linear connector spring characteristics was proposed. By introducing correction factors for individual segments of the load–slip curve, the modified spring model allows the full OPH material formulation to be retained without double counting plastic effects. The resulting numerical predictions show satisfactory agreement with experimental observations, confirming that the proposed stiffness-rescaling approach provided satisfactory agreement with the experimental response for the investigated configurations. However, the correction factors are configuration-specific calibration parameters and require independent validation before application to other connector layouts or full-scale TTC members.

The results presented in this study should be interpreted within the range of connection configurations investigated experimentally. The tested variants allowed the main mechanical effects associated with connector arrangement and stiffness to be identified; however, they do not cover the full range of possible design parameters, such as connector type, diameter, embedment depth or spacing. Consequently, the proposed analytical description and the corresponding adjustment parameters should not be directly extrapolated to substantially different configurations without additional verification. Further parametric studies are therefore required to extend the applicability of the proposed approach and to support the formulation of more general design recommendations. Such analyses are planned as a continuation of the present research. The numerical model should be interpreted as a calibrated representation of the investigated configurations within the experimentally observed load–slip range. Extrapolation beyond this range is not recommended without independent experimental validation. Future work will therefore focus on validation using full-scale TTC members and on parametric studies aimed at assessing the transferability of the proposed modelling approach.

## Figures and Tables

**Table 1:** Mechanical properties of LVL (nominal values in MPa, density in kg/m^3^) [19,20].

Material Parameters	LVL X	LVL R
Modulus of elasticity, parallel to grain, mean value *E*_0,*mean*_	10,600	14,000
Bending strength, flatwise, parallel to grain *f_m_*_,0,*flat*,*k*_	36.0	50.0
Tension strength, parallel to grain *f_t_*_,0,*k*_	22.0	36.0
Compression strength, parallel to grain *f_c_*_,0,*k*_	30.0	40.0
Density (characteristic value) *ρ_k_*	480.0	480.0

**Table 2:** Mechanical properties of GL28h (nominal values in MPa, density in kg/m^3^) [21].

Material Parameters	Value
Modulus of elasticity, parallel to grain, mean value *E*_0,*mean*_	12,600
Bending strength *f_m_*_,*k*_	28.0
Tension strength, parallel to grain *f_t_*_,0,*k*_	22.3
Compression strength, parallel to grain *f_c_*_,0,*k*_	28.0
Density (characteristic value) *ρ_k_*	425.0

**Table 3:** Push-out specimens.

Specimen	Slab	Beam	Screws
C_10_45.1	LVL X 45	200 × 300 (GL 28)	carpenter
C_10_45.2	LVL X 45	200 × 300 (GL 28)	carpenter
C_10_45.3	LVL X 45	200 × 300 (GL 28)	carpenter
H_10_45.1	LVL X 45	200 × 300 (GL 28)	hexagon head
H_10_45.2	LVL X 45	200 × 300 (GL 28)	hexagon head
H_10_45.3	LVL X 45	200 × 300 (GL 28)	hexagon head
C_10_75.1	LVL R 75	200 × 300 (GL 28)	carpenter
C_10_75.2	LVL R 75	200 × 300 (GL 28)	carpenter
C_10_75.3	LVL R 75	200 × 300 (GL 28)	carpenter
H_10_75.1	LVL R 75	200 × 300 (GL 28)	hexagon head
H_10_75.2	LVL R 75	200 × 300 (GL 28)	hexagon head
H_10_75.3	LVL R 75	200 × 300 (GL 28)	hexagon head

**Table 4:** The results of the push-out tests of the shear connections using carpenter screws and 45 mm LVL slabs (per one connector).

Parameter	Specimen			Mean	SD	COV [%]
	C_10_45.1	C_10_45.2	C_10_45.3			
*P_ult_* [kN]	12.9	14.6	15.0	14.2	1.12	7.87
*s_ult_* [mm]	37.4	41.7	45.1	41.4	3.86	9.32
*k_pre_* [kN/mm]	0.96	1.06	1.05	1.02	0.05	5.20
*k*_0.6_ [kN/mm]	0.65	0.71	0.57	0.64	0.07	11.40

**Table 5:** The results of the push-out tests of the shear connections using hexagon-head wood screws and 45 mm LVL slabs (per one connector).

Parameter	Specimen			Mean	SD	COV [%]
	H_10_45.1	H_10_45.2	H_10_45.3			
*P_ult_* [kN]	18.5	19.7	17.8	18.6	0.96	5.15
*s_ult_* [mm]	24.5	22.1	20.3	22.3	2.11	9.45
*k_pre_* [kN/mm]	7.25	7.14	9.19	7.86	1.16	14.70
*k*_0.6_ [kN/mm]	1.99	1.76	2.81	2.19	0.55	25.20

**Table 6:** The results of the push-out tests of the shear connections using carpenter screws and 75 mm LVL slabs (per one connector).

Parameter	Specimen			Mean	SD	COV [%]
	C_10_75.1	C_10_75.2	C_10_75.3			
*P_ult_* [kN]	14.9	13.7	14.9	14.5	0.69	4.78
*s_ult_* [mm]	44.0	43.2	43.9	43.7	0.44	1.00
*k_pre_* [kN/mm]	0.74	0.73	0.85	0.77	0.06	8.30
*k*_0.6_ [kN/mm]	0.53	0.58	0.59	0.57	0.03	5.10

**Table 7:** The results of the push-out tests of the shear connections using hexagon-head wood screws and 75 mm LVL slabs (per one connector).

Parameter	Specimen			Mean	SD	COV [%]
	H_10_75.1	H_10_75.2	H_10_75.3			
*P_ult_* [kN]	19.7	18.3	24.1	20.7	3.03	14.62
*s_ult_* [mm]	29.0	26.1	37.1	30.7	5.70	18.55
*k_pre_* [kN/mm]	3.19	2.67	3.64	3.17	0.49	15.30
*k*_0.6_ [kN/mm]	1.29	1.10	1.03	1.14	0.13	11.80

**Table 8:** Comparison between experimental connector capacities and predictions obtained from Equation (1).

Configuration	Nominal Screw Diameter Used in Equation (1), *d* [mm]	*P_pred_* [kN/Connector]	*P_exp_*_,*mean*_ [kN/Connector]	*P_exp_*/*P_pred_*
C_10_45	10	16.2	14.2	0.88
C_10_75	10	16.2	14.5	0.90
H_10_45	10	16.2	18.6	1.15
H_10_75	10	16.2	20.7	1.28

Note: *d* denotes the nominal screw diameter used in Equation (1) and not the predrilled-hole diameter. *P_pred_* is the reference analytical capacity obtained from Equation (1) and should not be confused with the specimen-specific *F_est_* used to define the experimental loading sequence.

**Table 9:** Mechanical properties of materials used in FEM.

Parameter Units	*E*_1_ [MPa]	*E*_2_ [MPa]	*E*_3_ [MPa]	*ν*_12_ [-]	*ν*_13_ [-]	*ν*_23_ [-]	*G*_12_ [MPa]	*G*_13_ [MPa]	*G*_23_ [MPa]	*f* [MPa]	*ε_pl_* [-]
LVL X [19,27]	13,400	700	700	0.48	0.48	0.22	900	900	90	22.0	0.0
LVL R [20,27]	17,400	500	500	0.48	0.48	0.22	1070	1070	100	36.0	0.0
GL [28,29]	11,439	732	458	0.34	0.36	0.42	715	529	690	19.2	0.0

**Table 10:** Hill yield function parameters [28,29,30,31].

Parameter	*R*11	*R*22	*R*33	*R*12	*R*13	*R*23
LVL X	1.000	0.182	0.182	0.362	0.362	0.362
LVL R	0.800	0.100	0.100	0.221	0.221	0.221
GL	1.000	0.141	0.141	0.316	0.316	0.316

**Table 11:** The adopted parameters describing the tri-linear mechanical model for an individual connector.

Segment		Type			
		C_10_45	C_10_75	H_10_45	H_10_75
ab	slip range [mm]	0.00–6.84	0.00–5.84	0.00–1.24	0.00–2.13
	*k*_ab_ [kN/mm]	1.00	1.06	7.14	3.85
bc	slip range [mm]	6.84–34.39	5.84–42.90	1.24–17.57	2.13–26.15
	*k*_bc_ [kN/mm]	0.29	0.22	0.55	0.48
cd	slip range [mm]	34.39–50.00	42.90–50.00	17.57–30.00	26.15–30.00
	*k*_cd_ [kN/mm]	0.0	0.0	0.0	0.0

**Table 12:** Stiffness correction factors for spring FE modelling.

No.	Stiffness Correction Factor *β*_ij_	C_45	C_75	H_45	H_75
1	*β*_ab_	1.50	1.33	3.47	1.94
2	*β*_bc_	2.00	1.71	3.74	3.36

## Data Availability

The original contributions presented in this study are included in the article. Further inquiries can be directed to the corresponding author.

## References

[1] Esmaeildoust S., Tomlinson D., Chui Y.H. (2025). Performance of Timber-Concrete Composite (TCC) Systems Connected with Inclined Screws: A Literature Review. J. Compos. Sci..

[2] Bedon C., Sciomenta M., Fragiacomo M. (2021). Correlation Approach for the Push-Out and Full-Size Bending Short-Term Performances of Timber-to-Timber Slabs with Self-Tapping Screws. Eng. Struct..

[3] Bedon C., Sciomenta M., Fragiacomo M. (2020). Mechanical Characterization of Timber-to-Timber Composite (TTC) Joints with Self-Tapping Screws in a Standard Push-Out Setup. Appl. Sci..

[4] Studziński R., Ciesielczyk K. (2019). Connection Stiffness between Thin-Walled Beam and Sandwich Panel. J. Sandw. Struct. Mater..

[5] Chuda-Kowalska M., Gajewski T., Garbowski T. (2015). Mechanical Characterization of Orthotropic Elastic Parameters of a Foam by the Mixed Experimental-Numerical Analysis. J. Theor. Appl. Mech..

[6] Chiniforush A.A., Valipour H.R., Ataei A. (2021). Timber-Timber Composite (TTC) Connections and Beams: An Experimental and Numerical Study. Constr. Build. Mater..

[7] Sciomenta M., Gualtieri P., Spera L., Contu F., Fragiacomo M. (2024). Timber-Timber Composite (TTC) Joints Made of Short-Supply Chain Beech: Push-Out Tests of Inclined Screw Connectors. Mater. Struct..

[8] Patrizi N., Sporchia F., Ruini A., Neri E., Bruno M., Zarroli G., Bastianoni S., Marchettini N. (2026). Designing for Reuse in Timber Buildings: The Environmental Benefits of Aligning the Time of Nature and Time of Use. Resour. Conserv. Recycl..

[9] Ottenhaus L.-M., Yan Z., Brandner R., Leardini P., Fink G., Jockwer R. (2023). Design for Adaptability, Disassembly and Reuse-A Review of Reversible Timber Connection Systems. Constr. Build. Mater..

[10] Nie Y., Valipour H.R. (2021). Experimental and Numerical Study of Long-Term Behaviour of Timber-Timber Composite (TTC) Connections. Constr. Build. Mater..

[11] Sun X., He M., Li Z., Ou J., Zheng X., Wei M. (2025). Experimental Study on the Long-Term Creep Behavior of Cross-Laminated Timber under Axial Compression Used for Mass Timber Structural Systems. Eng. Struct..

[12] Zheng X., Li Z., He M., Lam F. (2021). Experimental Investigation on the Rheological Behavior of Timber in Longitudinal and Transverse Compression. Constr. Build. Mater..

[13] Dubois F., Dopeux J., Pop O., Metrope M. (2023). Long-Term Creep Behavior of Timber Columns: Experimental and Numerical Protocols. Eng. Struct..

[14] Chiniforush A.A., Valipour H.R., Bradford M.A., Akbarnezhad A. (2019). Long-Term Behaviour of Steel-Timber Composite (STC) Shear Connections. Eng. Struct..

[15] Jiang Y., Hong W., Hu X., Crocetti R., Wang L., Sun W. (2017). Early-Age Performance of Lag Screw Shear Connections for Glulam-Lightweight Concrete Composite Beams. Constr. Build. Mater..

[16] (2004). Eurocode 2: Design of Concrete Structures-Part 1-1: General Rules and Rules for Buildings.

[17] (2004). Eurocode 5: Design of Timber Structures-Part 1-1: General-Common Rules and Rules for Buildings.

[18] Jelušič P., Kravanja S. (2018). Flexural Analysis of Laminated Solid Wood Beams with Different Shear Connections. Constr. Build. Mater..

[19] STEICO (2026). Declaration of Performance No. 03-0006-04.

[20] STEICO (2026). Declaration of Performance No. 03-0008-04.

[21] (2013). Timber Structures-Glued Laminated Timber and Glued Solid Timber.

[22] (2018). European Technical Assessment.

[23] Hassanieh A., Valipour H., Bradford M. (2016). Load-Slip Behaviour of Steel-Cross Laminated Timber (CLT) Composite Connections. J. Constr. Steel Res..

[24] (1991). Timber Structures-Joints Made with Mechanical Fasteners-General Principles for the Determination of Strength and Deformation Characteristics.

[25] Hassanieh A., Valipour H.R., Bradford M.A. (2016). Experimental and Analytical Behaviour of Steel-Timber Composite Connections. Constr. Build. Mater..

[26] (2004). Eurocode 4: Design of Composite Steel and Concrete Structures-Part 1-1: General Rules and Rules for Buildings.

[27] Abramowicz M., Chybiński M., Polus Ł., Wróblewski T. (2024). Free Vibrations of Sustainable Laminated Veneer Lumber Slabs. Sustainability.

[28] Kawecki B. (2021). Dobór Parametrów Modeli Obliczeniowych Pełnych Dźwigarów Z Kompozytów Drewno-polimerowych Zbrojonych Włóknami.

[29] Ciesielczyk K., Studziński R., Chybiński M., Ścigałło J., Szymkuć W., Grabski J.K., Marciniak T., Polus Ł. (2025). An Assessment of the Behaviour of Ceiling Beams of Different Structure. Appl. Sci..

[30] Dassault Systèmes (2025). Abaqus 2025 Online Documentation. https://www.3ds.com/support/documentation.

[31] Chybiński M., Polus Ł., Zieliński M. (2025). Short-Term Behaviour of Laminated Veneer Lumber Slabs Subjected to Bending. Adv. Sci. Technol. Res. J..

[32] Hill R. (1948). A Theory of the Yielding and Plastic Flow of Anisotropic Metals. Proc. R. Soc. Lond. A.

[33] Hong J.-P., Barrett J.D. (2010). Three-dimensional finite-element modeling of nailed connections in wood. J. Struct. Eng..

[34] Hong J.-P., Barrett J.D., Lam F. (2011). Three-dimensional finite element analysis of the Japanese traditional post-and-beam connection. J. Wood Sci..

